# Tuning the electrical properties of the heart by differential trafficking of K_ATP_ ion channel complexes

**DOI:** 10.1242/jcs.141440

**Published:** 2014-05-01

**Authors:** Eric C. Arakel, Sören Brandenburg, Keita Uchida, Haixia Zhang, Yu-Wen Lin, Tobias Kohl, Bianca Schrul, Matthew S. Sulkin, Igor R. Efimov, Colin G. Nichols, Stephan E. Lehnart, Blanche Schwappach

**Affiliations:** 1 Department of Molecular Biology, Center for Biochemistry and Molecular Cell Biology, Heart Research Center Göttingen, University Medicine Göttingen, Humboldtallee 23, 37073 Göttingen, Germany; 2 Faculty of Life Sciences, University of Manchester, Oxford Road, Manchester M13 9PT, UK; 3 Department of Cardiology & Pulmonology, Heart Research Center Göttingen, University Medicine Göttingen, Robert-Koch-Straße 40, 37075 Göttingen, Germany; 4 Department of Cell Biology and Physiology, and Center for the Investigation of Membrane Excitability Diseases, Washington University School of Medicine, 660 S. Euclid Avenue, Box 8228, St. Louis, MO 63110, USA; 5 Max-Planck Institute for Biophysical Chemistry, D-37077 Göttingen, Germany; 6 Department of Biomedical Engineering, Washington University, St. Louis, MO 63110, USA; 7 Center for Biomedical Engineering and Technology, University of Maryland Baltimore, Baltimore, MD 21201, USA

**Keywords:** ATP-sensitive K^+^ channels, COPI, K_ATP_, PKA, Trafficking, Protein kinase A, Cardiomyocyte, 14-3-3, Coatomer, Arg-based retrieval signal

## Abstract

The copy number of membrane proteins at the cell surface is tightly regulated. Many ion channels and receptors present retrieval motifs to COPI vesicle coats and are retained in the early secretory pathway. In some cases, the interaction with COPI is prevented by binding to 14-3-3 proteins. However, the functional significance of this antagonism between COPI and 14-3-3 in terminally differentiated cells is unknown. Here, we show that ATP-sensitive K^+^ (K_ATP_) channels, which are composed of Kir6.2 and SUR1 subunits, are stalled in the Golgi complex of ventricular, but not atrial, cardiomyocytes. Upon sustained β-adrenergic stimulation, which leads to activation of protein kinase A (PKA), SUR1-containing channels reach the plasma membrane of ventricular cells. We show that PKA-dependent phosphorylation of the C-terminus of Kir6.2 decreases binding to COPI and, thereby, silences the arginine-based retrieval signal. Thus, activation of the sympathetic nervous system releases this population of K_ATP_ channels from storage in the Golgi and, hence, might facilitate the adaptive response to metabolic challenges.

## INTRODUCTION

Hormone signaling rapidly adapts the function of cells to the physiological requirements of the organism. Regulated translocation of ion channels and transporters to the plasma membrane is one important mechanism of the cellular response. Prominent examples include insulin-triggered GLUT4 translocation (Bogan, 2012) and growth-hormone-induced translocation of TRPC5 channels (Abe and Puertollano, 2011; Bezzerides et al., 2004). Specialized post-Golgi storage vesicles and endosomal membranes contribute to the storage, rapid exposure and recycling of such cargo proteins, but the extent of the participation of the early secretory pathway in the regulated deployment of membrane proteins is unknown. Here, we consider the metabolically-sensitive ATP-sensitive K^+^ (K_ATP_) channel as an example of a heteromultimeric cargo protein that is stored in, and released from, the Golgi compartment upon hormone-induced signal transduction.

K_ATP_ channels are hetero-octameric multimers of four pore-forming Kir6.1 (*KCNJ8*) or Kir6.2 (*KCNJ11*) subunits and four sulfonylurea receptor [SUR1 (*ABCC8*) or SUR2 (*ABCC9*)] subunits (Nichols, 2006). Coexpression of the two types of subunit is necessary to achieve functional expression of K_ATP_ channels (Tucker et al., 1997) through a checkpoint mechanism (Zerangue et al., 1999) – the exposure of arginine (Arg)-based ER retention and retrieval motifs by Kir6.2 and SUR1 prevents cell surface transport unless stoichiometrically assembled hetero-octamers are formed. Subsequent work has identified the COPI complex as the vesicle coat involved in the recognition of Arg-based signals (Michelsen et al., 2007) and 14-3-3 proteins as a cytosolic factor that facilitates efficient cell surface expression (Heusser et al., 2006). The latter finding coincided with the discovery that many ion channels and plasma membrane proteins strictly require 14-3-3 to reach the cell surface (Gödde et al., 2006; O'Kelly et al., 2002; Rajan et al., 2002; Smith et al., 2011). For such cargo proteins, the lack of an interaction with 14-3-3 leads to an accumulation of cargo in the Golgi compartment (Gödde et al., 2006; Zuzarte et al., 2009). Intriguingly, all cargo proteins that require 14-3-3 for cell surface expression also possess COPI-interaction motifs (Gödde et al., 2006; O'Kelly et al., 2002; O'Kelly and Goldstein, 2008; Shikano et al., 2005; Smith et al., 2011; Zuzarte et al., 2009), raising the possibility that the antagonism between COPI and 14-3-3 is a key control mechanism of Golgi trafficking. 14-3-3 proteins predominantly recognize phosphorylated client proteins and participate in signal transduction cascades (Morrison, 2009). Taken together, these facts evoke the hypothesis that cargo interactions with COPI and 14-3-3 might underlie physiologically regulated sorting events, in addition to providing a basic assembly checkpoint.

Native K_ATP_ channels are highly expressed in multiple tissues. In cardiac muscle cells, they couple electrical and metabolic signals at the cell surface during adaptation to stress (Zingman et al., 2002), hyperpolarizing the cells and preventing Ca^2+^ entry under conditions of energy depletion. Thus, they might offer protection from life-threatening heart damage during ischemia or sustained β-adrenergic stimulation, as demonstrated previously in mice that had genetic deletions of K_ATP_ channel subunits (Miki et al., 2002; Suzuki et al., 2002; Yamada et al., 2006; Zingman et al., 2002). Human K_ATP_ mutations, many of which affect the trafficking of the channel (Yan et al., 2007), underlie different K_ATP_ channelopathies and can substantially increase the risk for heart disease (Nichols et al., 2013). All four types of K_ATP_ subunits have been identified in the heart (Philip-Couderc et al., 2008), but expression varies from region to region and can change under pathophysiological conditions (Isidoro Tavares et al., 2009; Isidoro Tavares et al., 2007; Raeis-Dauvé et al., 2012). From the genetically tractable mouse heart, it is clear that SUR2A and Kir6.2 subunits are important components of ventricular K_ATP_ channels, whereas SUR1 and Kir6.2 subunits are crucial for atrial channels (Flagg et al., 2008). Of note, these two subunits also form the pancreatic K_ATP_ channel complex, which is essential for insulin secretion and is the molecular target of common anti-diabetic sulfonylureas. The cellular processes that control the molecular diversity of K_ATP_ channels in general and, specifically, in different heart tissues – such as atria and ventricles – is currently unknown. We, therefore, assessed K_ATP_ channel complex assembly, as well as the localization and vesicular trafficking of SUR subunits in different cardiac chambers. We describe the presence of SUR1 in both chambers of the heart – calling attention to the controversial notion that sulfonylureas increase cardiovascular risk in type II diabetic patients (Garratt et al., 1999; Goldner et al., 1971; Henry, 1998).

## RESULTS

### The assembly status and localization of K_ATP_ channels in cardiac myocytes

**Fig. 1.**
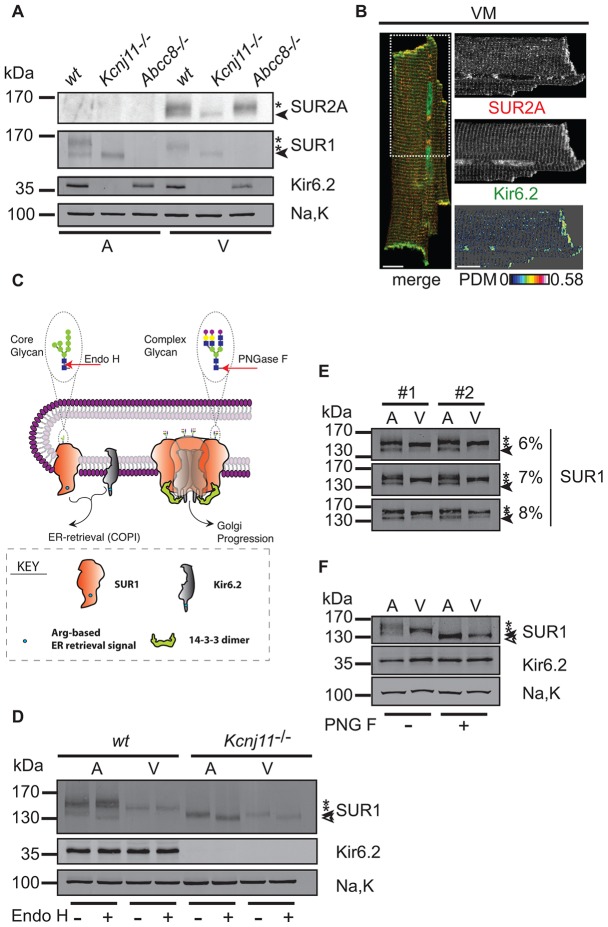
**Biochemical analysis of K_ATP_ channel subunits in atria and ventricles.** (A) Western blotting (see supplementary material Table S1 for antibodies) for SUR2A, SUR1, Kir6.2 and the α1 subunit of the Na^+^/K^+^-ATPase (Na,K) in membranes from mouse atrial (A) and ventricular tissue (V). Filled arrowheads and asterisks indicate core- and complex-glycosylated SUR proteins, respectively. The western blot is representative of three independent experiments. (B) Confocal analysis of immunostained mouse ventricular myocytes (VM). SUR2A (red) and Kir6.2 (green) signals are shown by the region of interest (ROI) that is indicated in the merged whole cell image (dashed white box). Kir6.2 nuclear staining is unspecific (see supplementary material Fig. S2 for knockout control). PDM denotes the product of differences from the mean, indicating colocalization by intensity correlation analysis (Li et al., 2004). Values of intensity correlation quotient between 0 and 0.5 indicate co-dependent staining and were 0.16±0.005 for VM (mean±s.e.m., *n* = 11). Scale bars: 10 µm. (C) Representation of SUR1 and Kir6.2 as cargo proteins of the early secretory pathway and the rationale of glycan analysis using Endo H and PNGase F. Shapes and symbols are identified by the boxed key. (D) Western blotting for SUR1, Kir6.2 and Na,K in membranes from atrial (A) and ventricular (V) tissues from wild-type or *Kcnj11*^-/-^ mice. Treatment with Endo H was as indicated; open arrowhead, filled arrowhead and asterisks mark deglycosylated, core-glycosylated, and complex-glycosylated forms of SUR1, respectively. Na,K serves as loading control. The western blot is representative of six independent experiments. (E) Western blotting for SUR1 in membranes from rat atrial (A) and ventricular (V) tissue demonstrating differing migration behaviors. The filled arrowheads indicate the core-glycosylated form of SUR1, and the asterisks denote the two complex-glycosylated forms of SUR1. The panel shows three technical replicates (the proteins were resolved on gels of increasing percentages – 6%, 7% and 8%) of two biological replicates (from two rats, #1 and #2). The western blot is representative of eight independent experiments. (F) Treatment with PNGase F glycosidase (PNG F) of solubilized membranes from rat atrial (A) or ventricular (V) tissue to probe whether differences in SUR1 migratory behavior (asterisks) were caused by differential complex glycosylation. The difference in migration was lost upon deglycosylation (indicated by the open arrowhead). The filled arrowhead indicates the core-glycosylated form of SUR1, and the asterisks indicate the complex-glycosylated forms of SUR1. The western blot is representative of eleven independent experiments.

We studied SUR1 and SUR2A in total membrane extracts from the dissected hearts of wild-type, *Kcnj11^−/−^* (Kir6.2 knockout) and *Abcc8^−/−^* (SUR1 knockout) mice (Fig. 1A; supplementary material Fig. S1A). SUR1 was expressed in both atria and ventricles, but SUR2A was absent from atria (see supplementary material Fig. S1B for quantification). Confocal image sections confirmed previous observations that had been obtained by scanning ion conductance microscopy (Korchev et al., 2000) that, in ventricular myocytes, SUR2A and Kir6.2 colocalized at the cell surface and at striations where transverse (T-)tubule membrane invaginations occur (Fig. 1B). The presence of SUR1 in ventricular myocytes (Fig. 1A) questions the concept that, in the ventricle, only SUR2A is associated with Kir6.2 (Babenko et al., 1998).

Both SUR1 and SUR2A are glycoproteins; SUR1 is N-glycosylated at positions Asn10 and Asn1050 (Conti et al., 2002), and sites for N-glycosylation are predicted at Asn9 and Asn330 of SUR2. We, therefore, employed glycosylation analysis to characterize trafficking of these K_ATP_ channel subunits within cardiac tissue (Fig. 1C). The glycosylation of secretory and membrane proteins occurs in different compartments of the secretory pathway because the modifying enzymes are confined to the endoplasmic reticulum (ER) or different regions of the Golgi (Kornfeld and Kornfeld, 1985). Hence, N-glycosylation status – i.e. the glycans and the extent of the modification – has been used to monitor the progression of such cargo proteins through the secretory pathway. Even without detailed analysis of the composition and length of the attached oligosaccharide, simple enzymatic tools can be used in combination with SDS-PAGE to assess changes in the electrophoretic mobility of cargo proteins, indicative of export from the ER and passage through the Golgi. Specifically, glycans added in the ER (core glycosylation) can be removed by Endoglycosidase H (Endo H), whereas the glycans added in the Golgi (complex glycosylation) are resistant to digestion with Endo H. Peptide-N-Glycosidase F (PNGase F) removes all types of N-glycosylation and can, thus, be used to demonstrate N-glycosylation *per se*.

In heterologous systems, cell surface expression of SUR proteins requires coexpression with Kir6.2 (or homologous Kir6.1) because COPI-dependent Arg-based ER-retrieval signals prevent the release of unassembled subunits from the early secretory pathway (Zerangue et al., 1999). Therefore, the glycosylation status reflects not only the steady-state localization of assembled complexes (the duration of passage through the Golgi, given that the degree of complex glycosylation is defined by the combined action of glycosyltransferases and glycosidases in the respective compartments) but also the assembly status of channel subunits (unassembled SUR proteins remain sensitive to Endo H).

Both SUR1 and SUR2A migrated faster and, hence, are presumably only core-glycosylated in the hearts of *Kcnj11^−/−^* mice (Fig. 1A), which suggests that complex-glycosylation of cardiac SUR1 and ventricular SUR2A depends on co-assembly with Kir6.2. Interestingly, in wild-type membranes, atrial and ventricular SUR1 was predominantly Endo-H-resistant and, therefore, complex-glycosylated (Fig. 1D). Concomitantly, SUR1 was sensitive to Endo H and, thus, only core-glycosylated in *Kcnj11^−/−^* hearts. This suggests that, in the heart, Kir6.2 is in both the atria and ventricles is the predominant assembly partner of SUR1. Co-assembly of SUR1 with Kir6.2 throughout the heart was also reflected by the decreased levels of cardiac Kir6.2 in *Abcc8^−/−^* mice (supplementary material Fig. S1C,D). SUR1 and Kir6.2 co-assemble in the brain, and the steady-state levels of either protein decreased upon knockout of the gene encoding the partnering subunit (supplementary material Fig. S1E). Hence, decreased levels of Kir6.2 in the absence of atrial or ventricular SUR1 (supplementary material Fig. S1C,D) is indicative of SUR1-containing K_ATP_ channels in both chambers.

Curiously, ventricular SUR1 was, consistently, a faster migrating Endo-H-resistant electrophoretic species compared with atrial SUR1, indicative of differential complex glycosylation (Fig. 1D,E). Treatment with PNGase F confirmed that SUR1 was complex-glycosylated in both chambers (Fig. 1F). Indeed, both atrial and ventricular SUR1 migrated more quickly and identically after treatment with PNGase F, confirming that the different electrophoretic mobility of atrial and ventricular SUR1 was due to differential complex glycosylation.

**Fig. 2.**
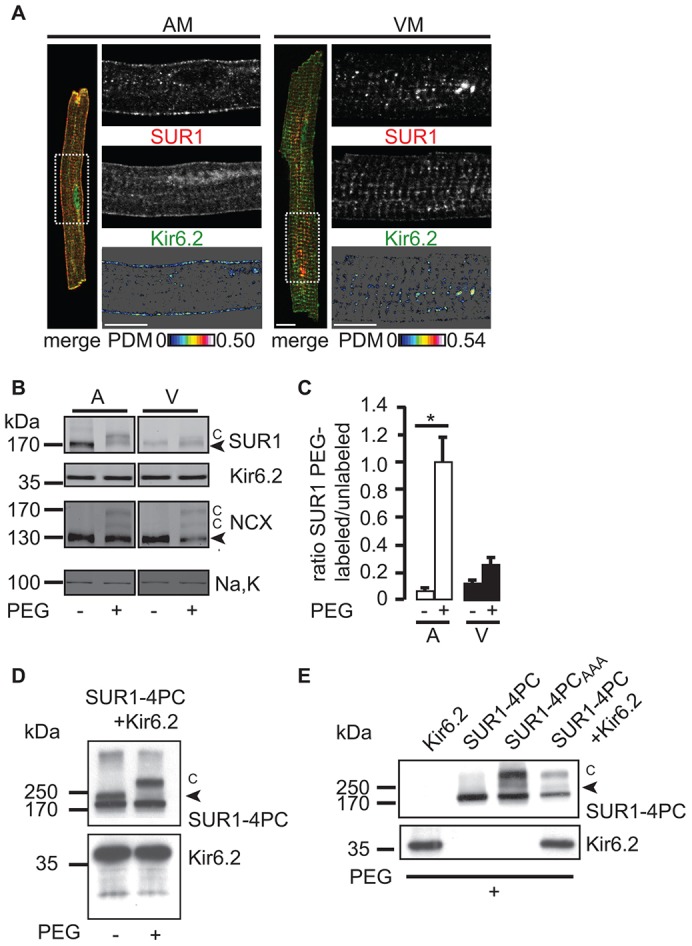
**SUR1–Kir6.2 K_ATP_ channels are localized differently in atrial and ventricular myocytes.** (A) Confocal analysis of immunostained mouse atrial (AM) or ventricular (VM) myocytes. SUR1 (red) and Kir6.2 (green) immunofluorescence signals are shown by the ROI as indicated in the whole cell image (merge, dashed white box). The values of intensity correlation quotient for AM were 0.20±0.007 (mean±s.e.m., *n* = 7) and VM 0.14±0.008 (mean±s.e.m., *n* = 4) confirming colocalization of Kir6.2 and SUR1 subunits. Scale bars: 10 µm. Refer to Fig. 8 for an illustration of cardiac myocyte morphology. (B) Cell surface PEGylation analysis in intact mouse hearts indicating cell surface expression of SUR1 in atrial, but not ventricular, myocytes. Modified bands are marked by ‘C’, the Na^+^/Ca^2+^ exchanger (NCX) is shown as a positive control for cell surface PEGylation. Kir6.2 and Na,K are not PEGylated and serve as negative controls. The western blot is representative of three independent experiments quantified in C. Note that the PEG modification is sensitive to reducing agents; hence, non-reducing conditions were employed in contrast with all other figures. Therefore, the core- and complex-glycosylated forms of SUR1 were not resolved. (C) The ratio of intensity of the labeled PEGylated SUR1 species (‘C’ in Fig. 2B) and unlabeled SUR1 (black arrowhead in Fig. 2B) for atria (open bars) and ventricles (filled bars). The increased PEGylation in atria was statistically significant (*P<0.05), whereas the ratio of PEGylated species was not significantly increased in ventricular membranes. (D) PEGylation of epitope-tagged SUR1-4PC (4× Protein C tags) and Kir6.2 in HEK293 cells. Western blotting for the PC-epitope is shown for PEG-maleimide treated sample and untreated control. ‘C’ indicates the position of PEGylated SUR1 species. The arrowhead indicates complex glycosylated SUR1. (E) SUR1 is only PEGylated upon coexpression with Kir6.2 or when the Arg-based retrieval signal is inactivated by site-directed mutagenesis (SUR1-4PC_AAA_), which is consistent with Arg-based retrieval signals preventing unassembled subunits from reaching the cell surface. The arrowhead indicates unmodified complex glycosylated SUR1.

Surprisingly, localization studies in isolated atrial and ventricular myocytes, using antibodies against SUR1 and Kir6.2 (the antibody specificity in the native cardiac environment using knockout controls for the respective antigen is shown in supplementary material Fig. S2A–C), revealed that SUR1-containing K_ATP_ channels were localized differently when atrial and ventricular myocytes were compared. In atrial myocytes, SUR1 and Kir6.2 colocalized at the plasma membrane (Fig. 2A, left panel); however, in ventricular myocytes, SUR2A was visible at the cell surface (Fig. 1B), but SUR1 did not localize at either the plasma membrane or in T-tubules. Instead, SUR1 was mostly retained in intracellular structures where it colocalized with Kir6.2 (Fig. 2A, right panel). We confirmed this difference in SUR1 surface localization between atria and ventricles by using a complementary biochemical method (Fig. 2B,C) – the labeling of cell-surface-exposed SUR1 by conjugating polyethylene glycol chains to extracellular cysteines (extracellular cysteine PEGylation). Fig. 2D,E demonstrates the specific labeling of only the complex-glycosylated form of SUR1 upon coexpression with Kir6.2 in HEK293 cells. The Na^+^/Ca^2+^ exchanger NCX1 is an established control protein for extracellular cysteine PEGylation in cardiac myocytes (Fig. 2B; Shen et al., 2007) and is present at ventricular T-tubules and the plasma membrane of atrial and ventricular myocytes (Jayasinghe et al., 2009). NCX1 was labeled with similar efficacy in both cell types, implying that all regions of the cell surface were accessible to the PEGylation reagent. By contrast, labeling of SUR1 was at least three times higher in atrial, compared with ventricular, myocytes (Fig. 2B,C), supporting the conclusion that more SUR1 is present at the plasma membrane of atrial than ventricular myocytes.

In *Kcnj11^−/−^* hearts, SUR1 was retained intracellularly in punctate structures throughout the cell but predominantly in a juxtanuclear compartment (inset, supplementary material Fig. S2A). Conversely, the cell surface of atrial *Abcc8^−/−^* myocytes was devoid of specific Kir6.2 staining, and all of the remaining Kir6.2 protein was detected inside of the cell (inset, supplementary material Fig. S2B). In ventricular *Abcc8^−/−^* myocytes, weak cell surface and striated Kir6.2 staining (presumably at T-tubules) was still visible (supplementary material Fig. S2B), which supports the hypothesis that SUR2A is the only partner subunit in the absence of SUR1 (Fig. 1A,B; supplementary material Fig. S1B). We, therefore, conclude that SUR1, which has functional properties distinct from SUR2A (Okuyama et al., 1998), assembles with Kir6.2 in atrial and ventricular myocytes; however, SUR1-containing K_ATP_ complexes are expressed at the cell surface of atrial myocytes but are predominantly intracellular in ventricular myocytes.

### The differential complex glycosylation of ventricular SUR reflect Golgi retention

**Fig. 3.**
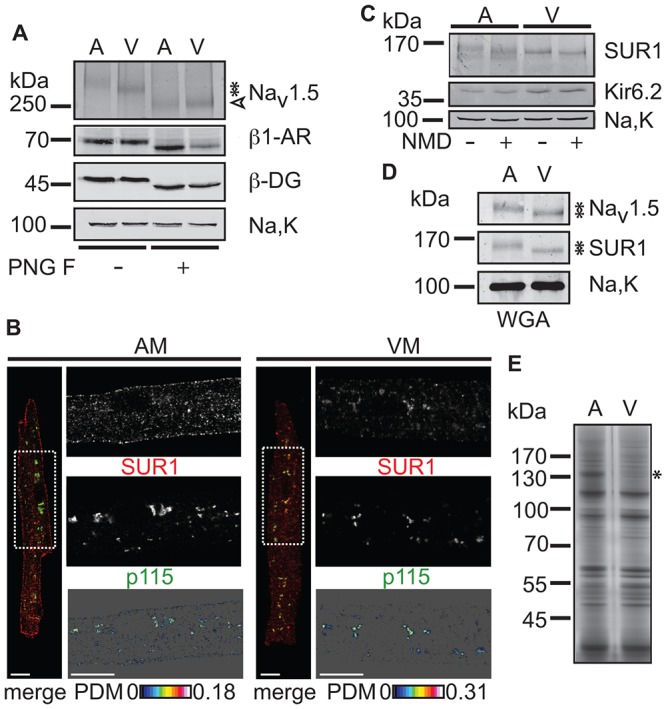
**SUR1–Kir6.2 K_ATP_ channels are retained in the Golgi of ventricular myocytes.** (A) Treatment with PNGase F glycosidase ‘PNG F’ of solubilized membranes from rat atrial (A) or ventricular (V) tissue. Like SUR1, the Na^+^ channel α-subunit Na_V_1.5 displayed differing migratory behavior (asterisks) between atria and ventricles, whereas the β-adrenergic receptor (β1-AR) and β-dystroglycan (β-DG) migration was indistinguishable in the two tissues. Na,K is not glycosylated and serves as a loading control. All three glycoproteins were fully deglycosylated by PNGase F (open arrowhead) leading to loss of the different migration patterns. The western blot is representative of three independent experiments. (B) Confocal analysis of immunostained mouse atrial (AM) or ventricular (VM) myocytes. SUR1 (red) and p115 (green) immunofluorescence signals are shown by the ROI in the whole cell image (merge, dashed white box). For an explanation of PDM analyses, see Fig. 1. Scale bars: 10 µm. Refer to Fig. 8 for an illustration of cardiac myocyte morphology. (C) Treatment of rat atrial and ventricular membranes with neuraminidase (NMD), as indicated, to probe for the extent of sialylation of SUR1. The western blot is representative of five independent experiments. (D) SDS eluates from a wheat germ agglutinin column (WGA) were probed with the indicated antibodies. Asterisks indicate the different migration of Na_V_1.5 and SUR1. The western blot is representative of three independent experiments. (E) Silver-stained eluates from WGA. The asterisk indicates a protein that was identified as peptidylglycine α-amidating monooxygenase, the staining of which was prominently different between atrial and ventricular tissue. The experiments shown in C,D,E were performed on rat cardiac tissue.

Having established that the faster electrophoretic migration of ventricular SUR1 (that was lost upon treatment with PNGase F, Fig. 1), and the lack of SUR1 at the ventricular myocyte surface (Fig. 2) could be the result of differential complex glycosylation, we assessed the electrophoretic mobility of other glycoproteins in atria and ventricles (Fig. 3A) – the cardiac Na^+^ channel Na_V_1.5 (Stocker and Bennett, 2006), the β1-adrenergic receptor (β1-AR) (Rohrer et al., 1996) and β-dystroglycan (β-DG) (Holt et al., 2000) were present in both atrial and ventricular membranes. Interestingly, atrial Na_V_1.5 exhibited a migratory shift similar to that of SUR1, whereas there was no observable difference in the migration of the β1-AR and β-DG glycoproteins between atria and ventricles. This suggests that the tissue-specific glycosylation profiles observed for SUR1 and Na_V_1.5 are restricted to only a subset of cargo proteins of the secretory pathway.

Colocalization analysis of SUR1 and the vesicle docking protein p115 (Nakamura et al., 1997), a marker of the cis-Golgi (Fig. 3B), showed that the Golgi were either juxtanuclear or scattered throughout both atrial and ventricular myocytes (Fawcett and McNutt, 1969; supplementary material Fig. S2D), and that the majority of ventricular SUR1-containing K_ATP_ channels was localized to the Golgi. Treatment with neuraminidase, which cleaves the glycosidic linkages of sialic acids, increased the electrophoretic mobility of atrial SUR1, which subsequently migrated close to the untreated ventricular form (Fig. 3C), indicating that atrial SUR1 is modified by sialic acid residues. Ventricular SUR1 was minimally affected by neuraminidase treatment, suggesting that both SUR1 forms carry the specific sugar moieties that are recognized by this exoglycosidase, but to different degrees. Because protein sialyltransferases are present in the medial and trans-Golgi compartments (Zhao et al., 2006), ventricular SUR1-containing K_ATP_ channels move at least as far as the medial Golgi, from where they are, presumably, retrieved due to recognition by the COPI-coat (Michelsen et al., 2007; Zerangue et al., 1999). Consistent with the differing electrophoretic mobility shifts upon treatment with neuraminidase, the atrial and ventricular forms of SUR1 and Na_V_1.5 bound wheat germ agglutinin (WGA), which specifically binds to *N-*acetylglucosamine, and neuraminic and sialic acids (Fig. 3D). Notably, the complement of proteins that was eluted from the WGA matrix was similar between atrial and ventricular proteins (Fig. 3E), suggesting that only a subset of WGA-binding proteins have varying glycosylation profiles. Thus, the differences in the migratory behaviors of SUR1 and Na_V_1.5 might reflect different residence times in the medial and trans-Golgi compartments, due to retrieval of these select cargo proteins. We conclude that Kir6.2–SUR1 complexes are retained within the Golgi of ventricular, but not atrial, myocytes, and that Na_V_1.5 is an independent and functionally essential cargo protein that is differentially glycosylated according to whether it is expressed in atria or ventricles. Thus, the secretory pathways of atrial and ventricular myocytes might differ in their control of the trafficking of membrane proteins besides K_ATP_.

### Channel deployment in response to β-adrenergic stimulation

**Fig. 4.**
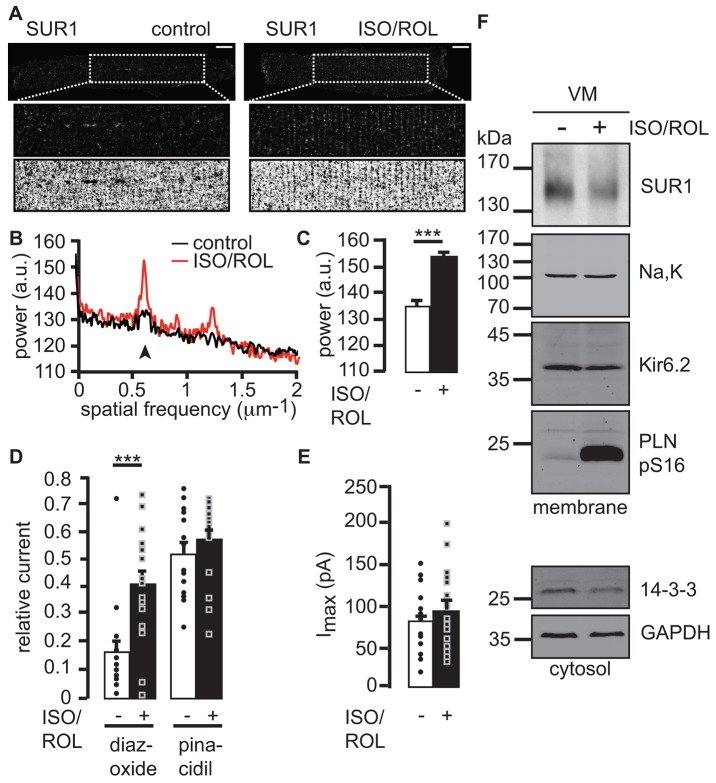
**β-adrenergic stimulation deploys Golgi-stalled SUR1–Kir6.2 channels to T-tubule membrane invaginations at striations of ventricular myocytes.** (A) Confocal analysis of mouse ventricular myocytes that were immunostained for SUR1 in the absence or presence of 10 µM isoproterenol and 10 µM rolipram (ISO/ROL). Dashed boxes indicate the magnified (2×) intracellular ROI (middle) and the binary inverse contrasted signal (bottom). Scale bars: 10 µm. Refer to Fig. 8 for a summary of cardiac myocyte morphology. (B) Power spectrum (Fourier analysis) of 20 untreated and 18 treated myocytes, the first peak indicates the degree of periodicity of the striated signal (arrowhead). (C) The average change in power at the first peak marked in B, error bars show the s.e.m., ***P<0.0005, a.u., arbitrary units. (D) Inside–out patch clamp recordings of mouse ventricular myocytes that were untreated or had been treated for 1 h and during recordings. ‘Relative current’ refers to the fraction of the current under conditions of no ATP that is activated by diazoxide or pinacidil. Individual data points are shown as circles (untreated) or squares (treated), ***P<0.0005, error bars reflect the s.e.m., *n* = 16 or 17 cells. (E) Inside–out patch clamp recordings of mouse ventricular myocytes that were untreated or had been treated with 10 µM isoproterenol and 10 µM rolipram each for 1 h and during recordings. I_max_, the maximum current under conditions lacking ATP. (F) Western blotting for SUR1, Na,K, Kir6.2 and the phosphorylated form of phospholamban (phosphorylated at serine residue 16, PLN pS16) in membranes (top panel), and 14-3-3 proteins and GAPDH in the cytosol (bottom panel) from mouse ventricular myocytes (VM) that were untreated or treated as in A–E (±ISO/ROL). The western blot is representative of three independent experiments.

In heterologous expression, SUR1-containing K_ATP_ channels are more sensitive to activation by Mg-nucleotide diphosphates (MgADP) than are SUR2A-containing channels (Masia et al., 2005). Hence, Golgi-retained SUR1-containing channels might provide a fully assembled pool of channels that, if subsequently trafficked to the cell surface, could provide enhanced metabolism-sensing and protection from the deleterious consequences of energy depletion. Kir6.2-containing K_ATP_ channels are known to contribute to the shortening of action potentials during catecholaminergic stress that is mediated by β-adrenergic receptors (Zingman et al., 2002), but the SUR composition of these channels is unknown. We applied the selective β-adrenergic agonist isoproterenol (ISO) with the cAMP-specific phosphodiesterase type 4 inhibitor rolipram (ROL) (Lehnart et al., 2005) to intact hearts for 1 h. In ventricular myocytes isolated from hearts that had been treated with ISO and ROL, we observed substantial spatial reorganization of the dispersed (Fig. 2A and Fig. 4A ‘control’) SUR1 signal into regular striation-associated fluorescence (Fig. 4A–C). This raises the possibility that SUR1-containing K_ATP_ channels are inserted into the cell surface membrane, particularly into T-tubule membranes. Notably, the spatial distribution of the Na^+^/Ca^2+^ exchanger NCX1, which localizes to the plasma membrane of ventricular myocytes within, and outside of, T-tubules, was not affected by sustained β-adrenergic stimulation (supplementary material Fig. S3A–C) but did overlap with SUR1 after treatment with ISO and ROL (supplementary material Fig. S3D). These findings are consistent with cAMP-dependent translocation of SUR1-containing K_ATP_ channels to T-tubules.

The K^+^ channel opener diazoxide potently activates SUR1-containing K_ATP_ channels but not SUR2A-containing channels, whereas pinacidil activates SUR2A- but not SUR1-containing channels (Flagg et al., 2008). Consistent with the above hypothesis, treatment with ISO and ROL significantly increased the diazoxide-sensitive component, but not the pinacidil-sensitive component, of the K_ATP_ channel current in wild-type ventricular myocytes that had been treated using the same protocol (Fig. 4D). The mean total K_ATP_ current was not significantly increased (Fig. 4E), although individual myocytes exhibited larger total K_ATP_ currents. The levels of SUR1, Kir6.2 and 14-3-3, as well as the phosphorylation status of phospholamban, a well-characterized target of protein kinase A (PKA, a major effector of β-adrenergic signal transduction) (Fig. 4F; see supplementary material Fig. S3E for quantification) confirmed the increased activation of β-adrenergic effectors and excluded increased amounts of total SUR1 or Kir6.2 as an explanation for the presence of SUR1-containing K_ATP_ channels at the ventricular myocyte surface.

### The abundance of 14-3-3 correlates with channel trafficking

**Fig. 5.**
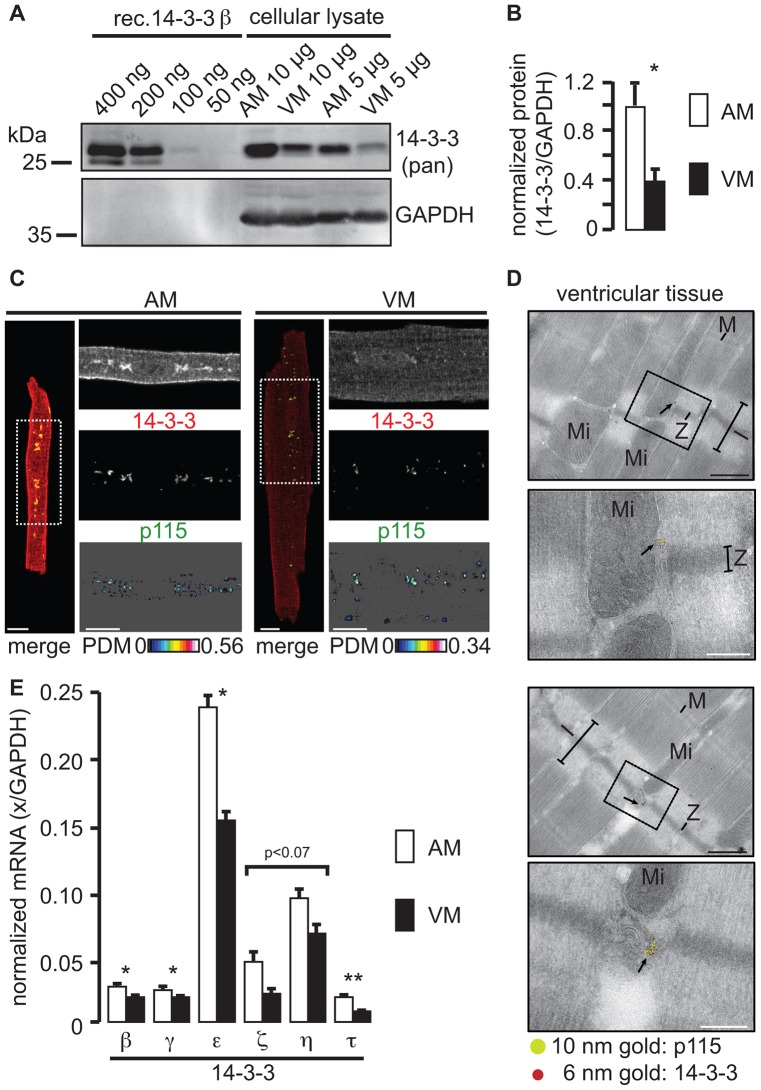
**Ventricular myocytes contain significantly lower amounts of 14-3-3.** Western blotting (A) and quantification (B) of three independent experiments using a pan-reactive antibody cocktail against 14-3-3 (supplementary material Table S1, antibodies 1b and 1c). The indicated amounts of soluble cellular lysate from rat atrial (AM) or ventricular (VM) myocytes were loaded. Varying concentrations of recombinant 14-3-3β (rec. 14-3-3 β) were used to approximate the detection threshold of the pan-reactive antibody. Glyceraldehyde-3-phosphate dehydrogenase (GAPDH) was detected as a loading control. *P<0.05. (C) Confocal analysis of immunostained mouse atrial (AM) or ventricular (VM) myocytes. 14-3-3 (red) and p115 (green) immunofluorescence signals are shown in the ROI indicated in the whole cell image (merge, dashed white box). Scale bars: 10 µm. Refer to Fig. 8 for an illustration of cardiac myocyte morphology. (D) Two different examples of immuno-electron microscopy that were performed on fixed cryosections from the left ventricle of mouse hearts. The upper picture indicates the inset that is shown in the lower panel. I, M and Z indicate the respective bands of the cardiac muscle. Mi, mitochondria and arrows point to the gold label (14-3-3, 6 nm and p115, 10 nm). Scale bars: 500 nm (black), 200 nm (white, the boxed inset magnification). (E) RT-PCR analysis of six 14-3-3 isoforms using mRNA from isolated rat atrial (AM) and ventricular (VM) myocytes. mRNA abundance is normalized to the message encoding GAPDH. The means were derived from three biological replicates. *P<0.05, **P<0.01, a non-significant value of *P*<0.07 is indicated for two isoforms.

K_ATP_ channels belong to a group of cargo proteins that recruit 14-3-3 proteins, yet where in the cell this occurs is unknown (Heusser et al., 2006). The 14-3-3-dependent cargo proteins TASK-1 (another K^+^ channel) and ADAM22 (a catalytically inactive metalloproteinase) both accumulate in cis- and medial-, but not trans-, Golgi compartments in the absence of an interaction with 14-3-3 (Gödde et al., 2006; Zuzarte et al., 2009), and we hypothesized that ventricular SUR1–Kir6.2 K_ATP_ channels have insufficient interaction with 14-3-3 proteins during intra-Golgi trafficking, which results in constitutive Golgi localization (Fig. 2) and reduced sialylation (Fig. 3). The levels of 14-3-3 protein were markedly lower in ventricular myocytes compared with those from atria (Fig. 5A,B), raising the possibility that the reduced availability of the 14-3-3 protein limits cell surface expression of SUR1–Kir6.2 K_ATP_ channels in ventricles. Immunofluorescence staining of atrial myocytes, by using an antibody that recognises all 14-3-3 isoforms, revealed strong labeling of juxtanuclear, submembraneous and intracellular compartments, including a weak striated pattern (Fig. 5C, AM). In ventricular myocytes, 14-3-3 immunostaining was much weaker (compare supplementary material Fig. S2D) and was primarily restricted to intracellular striations (Fig. 5C, VM), suggestive of a specific association with Z-lines and T-tubule junctions of the ER. In both atrial and ventricular myocytes, there was a substantial colocalization of 14-3-3 with p115 (Fig. 5C), which was adjacent to Z-lines of ventricular myocytes (Fig. 5D), suggesting that cardiac 14-3-3 proteins are present at the Golgi apparatus. This colocalization is consistent with previous observations that have shown overlapping immunofluorescence patterns for GM130, a p115-interacting Golgi matrix protein (Nakamura et al., 1997), and 14-3-3 in HeLa cells (Preisinger et al., 2004). Intriguingly, the localization of these Golgi elements in the vicinity of Z-lines coincides with the previous observation that coated vesicles are frequently observed in this region, but are only sometimes connected to the ER (Fawcett and McNutt, 1969). The colocalization of K_ATP_ channels with p115, and the different sialylation profiles of atrial and ventricular SUR1 (Fig. 3) suggests that the channel complexes are stalled in Golgi elements, possibly as part of a dedicated secretory pathway between the ER and T-tubules at Z-lines.

Six mammalian 14-3-3 isoforms were expressed in isolated myocytes, the most abundant were 14-3-3ε and 14-3-3η, but ventricular myocytes expressed every isoform to a lower extent compared with atrial myocytes (35–75% less mRNA) (Fig. 5E), this is consistent with the hypothesis that ventricular K_ATP_ channels are retained because of a reduced abundance of 14-3-3 isoforms. The results presented here raise the novel possibility that, in terminally differentiated cell types, such as cardiac myocytes, the availability of 14-3-3 proteins might be important in the regulation of the surface expression of specific cargoes, and that the availability of 14-3-3 proteins might underlie differences in the expression of functionally important cell surface proteins.

### Silencing of Arg-based signals by phosphorylation

**Fig. 6.**
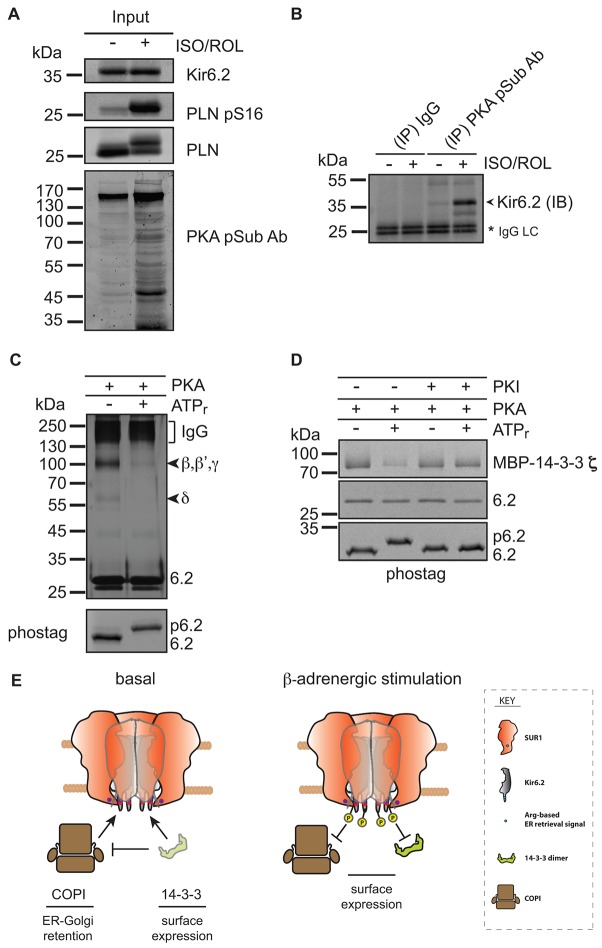
**Phosphorylation of the Kir6.2 C-terminus by PKA reduces COPI and 14-3-3 binding.** (A) Western blotting for Kir6.2, Phospholamban (PLN), a phosphorylated form of phospholamban (at serine residue 16, PLN pS16) and substrates phosphorylated by PKA (PKA pSub Ab) in hearts perfused in the presence (+) or absence (−) of 10 µM isoproterenol and 10 µM rolipram (ISO/ROL). (B) Phosphorylated Kir6.2 was immunoprecipitated (IP) using an antibody that binds PKA phosphorylated substrates (PKA pSub Ab). Solubilized membranes that had been prepared from treated (+) or untreated (−) hearts, as in A, were used. The blots were probed for Kir6.2 (IB, immunoblot). Purified Rabbit IgG (IgG) was used as a negative control. IgG LC refers to the antibody light chain and serves as a loading control. (C) Silver-stained eluates from a COPI binding assay reveals the reduction of COPI binding after phosphorylation of the C-terminus of Kir6.2. Compare supplementary material Fig. S4 for the quantification of three independent experiments. β, β', γ and δ refers to four subunits of coatomer. IgG refers to the co-eluted antibody from the IgG sepharose affinity matrix. ATPr, ATP regeneration system. The lower panel compares the electrophoretic mobility shift of the unphosphorylated (6.2) and phosphorylated (p6.2) Kir6.2 C-terminal peptide on a Phostag-polyacrylamide gel. (D) Coomassie-stained eluates from a 14-3-3 binding assay revealed a reduction of 14-3-3 binding after phosphorylation of the C-terminus of Kir6.2. The gel is representative of three independent experiments. PKI, protein kinase A inhibitor. (E) The release of SUR1-containing K_ATP_ channels from the antagonistic actions of COPI and 14-3-3 after ;phosphorylation.

We hypothesized further that a direct action of PKA induces cell surface trafficking of the SUR1–Kir6.2 complexes, in addition to its direct activating effects on channel function in heterologous overexpression systems (Béguin et al., 1999). Immunoprecipitation, by using an antibody that recognizes phosphorylated PKA-target motifs, strongly enriched Kir6.2 from solubilized membranes that had been prepared from mouse hearts treated with ISO and ROL, as compared with control hearts (Fig. 6A,B). Affinity purification of all of the phosphorylated proteins that were present in solubilized membranes, by using Phos-tag affinity chromatography (supplementary material Fig. S4A–C), confirmed that Kir6.2 was phosphorylated. Importantly, the antibody against PKA phosphorylated substrates blocked the binding of Kir6.2 to the Phos-tag affinity matrix from hearts that had been treated with ISO and ROL. Thus, we conclude that sustained β-adrenergic stimulation results in PKA-dependent phosphorylation of cardiac Kir6.2.

Next, we tested the possible consequences of PKA-mediated phosphorylation of Kir6.2 on channel trafficking by using an *in vitro* binding experiment to capture the inherently transient interaction with trafficking machinery, such as the COPI vesicle coat. Upon exposure of a peptide comprising the C-terminal 36 amino acids of Kir6.2 to the catalytic subunit of PKA, the peptide was phosphorylated (supplementary material Fig. S4D). The same activity was confirmed for endogenous PKA in cardiac cytosol and total membranes (supplementary material Fig. S4E), demonstrating that cardiac PKA forms can target the C-terminus of Kir6.2. Interestingly, phosphorylation of the Kir6.2 C-terminal peptide strongly reduced the binding of both COPI and 14-3-3 (Fig. 6C,D; supplementary material Fig. S5). Recombinant channel assays have shown previously that this consensus PKA phosphorylation site (serine residue 372, Fig. S4A) can be phosphorylated (Béguin et al., 1999). Beguin and colleagues reported that phosphorylation of this site underlies PKA-mediated enhanced gating, whereas Lin and colleagues (Lin et al., 2000) have reported that PKA-dependent gating was unaffected by mutation at this site. Thus, we cannot exclude an effect of such phosphorylation on gating, but the present data clearly suggest a significant effect on trafficking.

Although many 14-3-3-binding sites depend upon the phosphorylation of a serine residue that is part of the consensus binding-motif, negative effects of the phosphorylation of flanking serine residues on the binding to 14-3-3 have been described for other proteins (Waterman et al., 1998). This suggests that PKA-phosphorylated channels might be released from COPI-dependent retrieval, no longer requiring binding to 14-3-3 for trafficking and, hence, exiting the Golgi, irrespective of 14-3-3 availability (Fig. 6E).

### SUR1-containing K_ATP_ channels contribute to action potential duration during sustained β-adrenergic stimulation

**Fig. 7.**
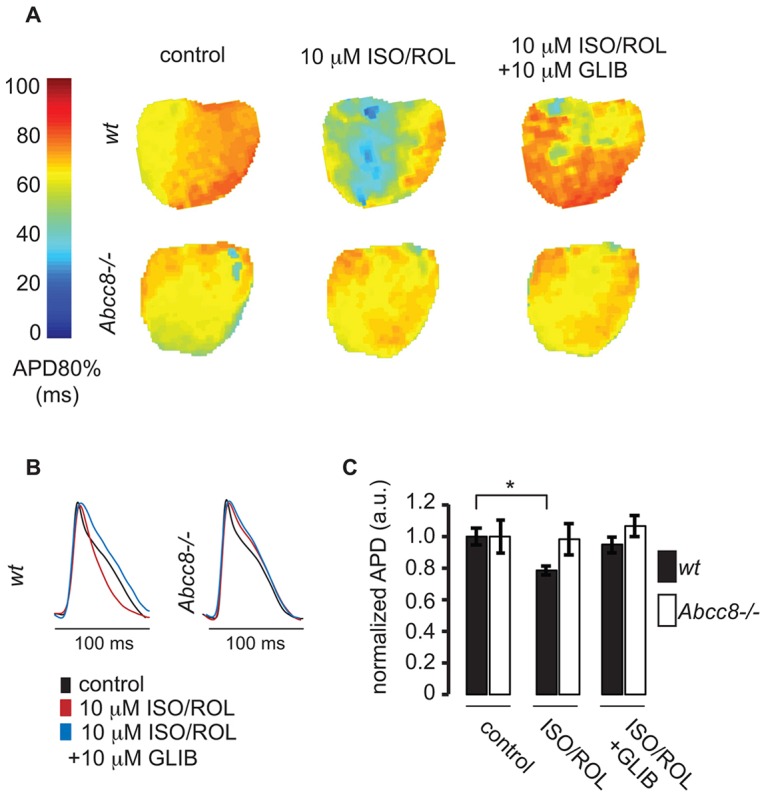
**Action potential shortening during sustained β-adrenergic stimulation requires SUR1.** (A) Representative APD maps from a wild-type (*wt*, top row) and *Abcc8^−/−^* (bottom row) ventricle were constructed from optical mapping recordings under control conditions (left column), 20 min after administration of 10 µM ISO and 10 µM ROL (center column), and 10 min after the addition of 10 µM glibenclamide (GLIB, right column). Each pixel in the map represents the APD80% from that region of the ventricular myocardium according to the color bar on the left. (B) The signal-averaged action potential traces from representative WT and *Abcc8^−/−^* ventricles are shown. (C) The normalized APD from WT hearts (*n* = 5) shows significant shortening (at 80% repolarization) upon treatment with ISO and ROL, and clear reversal with glibenclamide. However, the normalized APD from *Abcc8^−/−^* hearts (*n* = 5) shows no significant changes throughout the experiment. *P<0.05, means±s.e.m. a.u., arbitrary units.

K_ATP_ channel activation and decreased action potential duration (APD) can occur during sustained β-adrenergic stimulation (Zingman et al., 2002), but the SUR composition of these channels is unknown. In light of our observation that a ventricular population of SUR1-containing K_ATP_ channels can translocate to the T-tubule surface upon β-adrenergic signaling, we tested whether SUR1-containing channels might play a role in shortening of the APD. To this end, we performed optical mapping of action potentials in wild-type and *Abcc8^−/−^* hearts (Fig. 7A–C) under control conditions, after treatment with ISO and ROL, and after the same treatment in the presence of glibenclamide, which blunts the contribution of the K_ATP_ channel to shortening of the action potential (Zingman et al., 2002). Intriguingly, the APD in *Abcc8^−/−^* mice was unaffected upon treatment with ISO and ROL, or ISO and ROL in combination with glibenclamide, in contrast with wild-type hearts, where we confirmed the observations previously noted by Zingman and colleagues that β-adrenergic shortening of the APD required K_ATP_ channel activation (Zingman et al., 2002). This result delineates a physiological role for the PKA-regulated cell surface translocation of SUR1-containing channels during sustained β-adrenergic stimulation (Figs 4,6,7).

## DISCUSSION

**Fig. 8.**
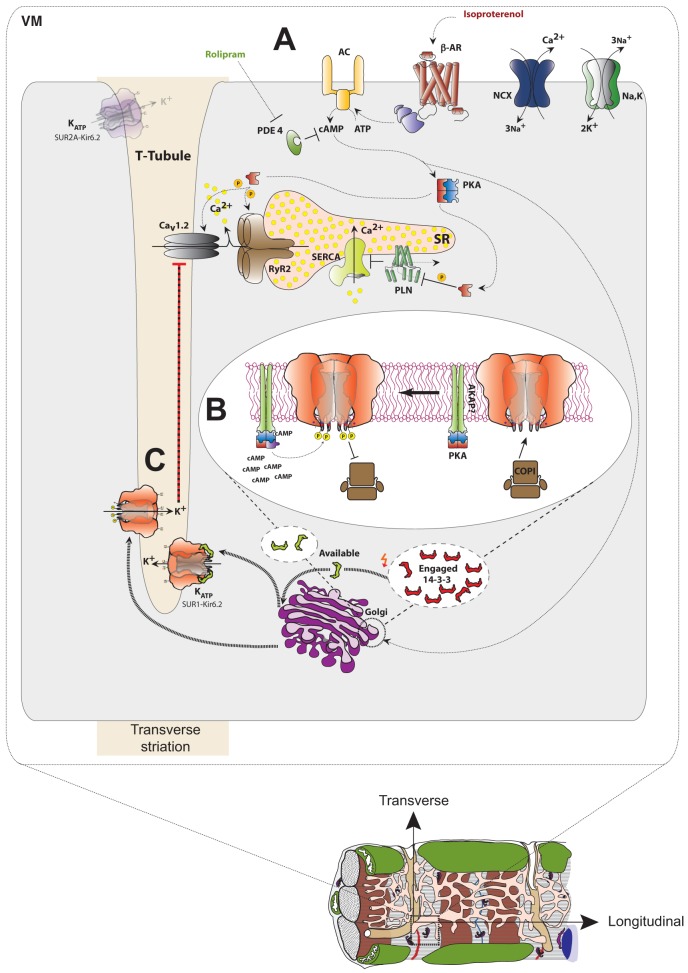
**Model of the regulated deployment of SUR1–Kir6.2 K_ATP_ channels in ventricular myocytes.** (A) Agonist (isoproterenol) binding to β -adrenergic receptors (β-AR) triggers the activation of the adenylyl cyclase (AC) through a β-AR-coupled G-protein, resulting in elevation of cAMP and the activation of PKA. Attenuation of signal transduction through degradation of cAMP by phosphodiesterase (PDE) was inhibited using the PDE4-specific inhibitor rolipram. Known PKA targets include the voltage-gated Ca^2+^ channel (Ca_V_1.2) and the Ryanodine receptor (RyR2), culminating in elevation of cytosolic Ca^2+^ [by release from the sarcoplasmic reticulum (SR) Ca^2+^ stores and by influx of extracellular Ca^2+^]. The phosphorylation of phospholamban (PLN) by PKA relieves its inhibitory effect on the SR Ca^2+^ pump (SERCA). For simplicity, the relevant PKA holoenzyme has been depicted as being cytosolic and not membrane associated. (B) PKA dependent phosphorylation of S372 (adjacent to the Arg-based ER retrieval signal) in Kir6.2 releases Golgi-stored SUR1–Kir6.2 K_ATP_ channels from COPI binding, thus, facilitating Golgi exit. An unknown kinase anchoring protein (AKAP), conceivably, localizes PKA to the vicinity of K_ATP_ channels. (C) Deployment of SUR1-containing K_ATP_ channels from the Golgi to the T-tubular plasma membrane. Hypothetically, signal transduction might affect the available pool of 14-3-3 proteins, in addition to direct phosphorylation of cargo proteins, by shifting the equilibrium between an engaged (substrate bound) and available (substrate free) pool, thus, overcoming the limitations of cell surface expression of 14-3-3 substrates by the limited availability of 14-3-3 proteins.

Our experiments provide novel insights into the cellular control of the localization and trafficking of an important ion channel complex in terminally differentiated cardiac myocytes (Fig. 8). SUR1-containing K_ATP_ channels constitutively reach the cell surface in only atrial myocytes (Fig. 2), potentially, because of the high abundance of 14-3-3 proteins (Fig. 5), which are required to overcome the COPI-dependent retrieval signals present in Kir6.2 and SUR1 (Heusser et al., 2006; Michelsen et al., 2005; Yuan et al., 2003). By contrast, SUR1-containing K_ATP_ channels are stalled in the Golgi of ventricular myocytes but are deployed to the cell surface upon sustained β-adrenergic stimulation (Figs 2–4). Interestingly, SUR2A-containing channels constitutively reach the plasma membrane in ventricular myocytes, despite the low abundance of 14-3-3 proteins (Figs 1, 5). Based on the consensus of Arg-based signals, SUR2 contains a less potent ER retrieval signal (RKQ) than SUR1 (RKR) (Konstas et al., 2002; Michelsen et al., 2005; Zerangue et al., 2001), possibly rendering SUR2A-containing K_ATP_ channels less dependent on 14-3-3. Upon phosphorylation of the C-terminus of Kir6.2, both COPI and 14-3-3 ceased to interact with the protein (Fig. 6; supplementary material Figs S4, S5), potentially, releasing the channel from COPI- and 14-3-3-dependent control of anterograde trafficking. Importantly, newly integrated SUR1-containing K_ATP_ channels in the T-tubule surface will be intrinsically more sensitive to metabolic activation than SUR2A containing channels (Masia et al., 2005) and are, probably, highly active due to phosphorylation by PKA (Béguin et al., 1999). Here, we identify a physiological role for this SUR1-containing channel population in action potential shortening during sustained β-adrenergic stimulation (Fig. 7). Our results are consistent with previous reports that have demonstrated the contribution of K_ATP_ channels to action potential shortening under these conditions (Zingman et al., 2002) but clarify the K_ATP_ channel subunits that are involved and indicate that the phenomenon relies on PKA-regulated deployment of SUR1-containing channels to the ventricular cell surface in T-tubules.

Our results identify a novel molecular mechanism that utilizes COPI-dependent storage in the Golgi for the regulated cell surface expression of a key cargo protein. Strikingly, most of the characterized Arg-based retrieval signals are flanked by serine residues, some of which are known targets of phosphorylation (supplementary material Table S2). Thus, interaction with COPI coat proteins might – in addition to providing an assembly checkpoint – be harnessed in terminally differentiated cell types to allow regulated deployment from the Golgi to the cell surface. Therefore, COPI-dependent storage and COPI-regulated deployment might explain conflicting results that implicate the activity of Arg-based signals in retrieval, as well as exit, from the early secretory pathway (compare references in supplementary material Table S2). We suggest a mechanism as to how SUR1-containing K_ATP_ channels could be released from COPI- and 14-3-3-dependent control and, hence, play a previously unrecognized role in the cAMP-dependent ‘fight-or-flight’ response of the heart (Figs 7, 8). SUR1-containing channels are very sensitive to blockade by sulfonylureas, lending additional weight to clinical recommendations that call for the re-evaluation of the cardiac risk that is associated with treatment with sulfonylurea in type II diabetes (Gore and McGuire, 2011; Schramm et al., 2011).

## MATERIALS AND METHODS

### Mice

Male wild-type, Kir6.2 knockout (*Kcnj11^−/−^*; described previously by Miki et al., 1998) and sulfonylurea receptor type-1 (SUR1) knockout (*Abcc8^−/−^*; described previously by Shiota et al., 2002) mice in the C57BL/6J background, aged 8–14 weeks, were used. All animal procedures were reviewed and approved by the Institutional Animal Care and Use Committees of the University Medical Center Göttingen and the Washington University School of Medicine in compliance with the humane care and use of laboratory animals.

### Cardiac tissue and myocyte preparation

Hearts were retrogradely perfused by a modified Langendorff solution (NaCl 120.4 mM, KCl 14.7 mM, KH_2_PO_4_ 0.6 mM, Na_2_HPO_4_ 0.6 mM, MgSO_4_ 1.2 mM, Na-HEPES 10 mM, NaHCO_3_ 4.6 mM, taurine 30 mM, 2,3-butanedione-monoxime 10 mM, glucose 5.5 mM, pH 7.4) for a period of 4 min at 37°C at a flow rate of 4 ml/min. For isolation of cardiomyocytes, the perfusion included collagenase type II (600 U/ml). The residual tissue was removed by using a 100-µm cell strainer (BD Falcon, 352360). Bovine calf serum (10%) and 12.5 µM CaCl_2_ in perfusion buffer was used to inhibit collagenase activity. Isolated myocytes were plated on laminin-coated glass coverslips at 1500 cells/cm^2^.

### Indirect immunofluorescence microscopy

Mouse atrial or ventricular myocytes were fixed with 4% paraformaldehyde (PFA), washed three times in PBS and incubated overnight in blocking buffer (10% bovine calf serum, 0.2% Triton X-100 in PBS). Primary antibodies were diluted (see supplementary material Table S1) in blocking buffer. Samples were incubated overnight at 4°C, washed three times in blocking buffer and incubated with Alexa-Fluorconjugated secondary antibodies (Invitrogen) for 2 h at room temperature.

### Image acquisition and colocalization analysis

All images were acquired by using a confocal microscope (Zeiss LSM 710, Jena, Germany) with the Plan-Apochromat 63×/1.40 Oil DIC M27 objective. All images were analyzed by ImageJ software (imagej.nih.gov). Colocalization analysis was performed by applying an intensity correlation analysis (Li et al., 2004) on regions of interest (ROIs) to generate colocalization maps and the intensity correlation quotient. Positive values (0–0.5) indicated co-dependent staining.

### Fourier transform analysis

Immunostaining for SUR1 was performed on 20 untreated ventricular myocytes and 18 ventricular myocytes that had been treated with isoproterenol and rolipram from four hearts. Confocal sections were selected omitting cell nuclei. The T-tubule-associated transverse striation pattern was aligned with the image *y*-axis by virtual image rotation. Fast Fourier transformation was performed from equally sized ROIs (∼36 Z-lines) using ImageJ version 1.43u. The power of periodic frequencies along the image *x*-axis (longitudinal cell axis) was derived from the Fourier domain images (not shown). Binary images in Fig. 4A were obtained by using thresholding of the raw data images and visualizing the alteration in spatial signal organization upon β-adrenergic stimulation.

### Immuno-transmission electron microscopy

Mouse hearts were dissected and perfused for 4 min with perfusion buffer and, for fixation of cardiac tissue, followed by 5 min perfusion with 4% PFA in PBS pH 7.4. The left ventricles were fixed for an additional 2 h in 4% PFA in PBS at room temperature followed by fixation in 2% PFA in PBS overnight at 4°C. The fixed ventricles were cut into small blocks, infused with 2.3 M sucrose in PBS at 4°C overnight and mounted on metal pins in an orientation allowing sectioning in the longitudinal axes. Ultrathin (75-nm) longitudinal cryosections were prepared according to the Tokuyasu method (Tokuyasu, 1973). For immunolabeling, sections were blocked with 1% bovine serum albumin in TBS and incubated with an antibody against p115, followed by a secondary antibody against mouse IgG coupled to 10-nm gold (Aurion). Following 5 min of crosslinking using 1% glutaraldehyde in PBS, the sections were probed with an antibody against 14-3-3 and then a secondary antibody against mouse IgG that had been coupled to 6-nm gold (Aurion). Sections were contrasted with 0.4% (w/v) uranyl acetate in 2 M methylcellulose for 15 min on ice, embedded in the same solution and examined with a Phillips CM120 transmission electron microscope. Micrographs were acquired with a CCD camera (Megaview III, Olympus Soft Imaging Systems) and processed using iTEM software.

### mRNA analysis

Three rat hearts were used to extract RNA from atrial and ventricular myocytes. Total RNA was extracted by using the Trizol method (Invitrogen), it was then treated with DNase I (TURBO DNase, 2 U/µl; Invitrogen) and further purified by phenol, chloroform and isoamyl alcohol extraction and subsequent ethanol precipitation. cDNA was obtained by reverse transcription (qScript cDNA SuperMix, Quanta BioSciences). Quantitative real-time (RT)-PCR was performed using an iQ5 cycler (Bio-Rad) and PerfeCTa SYBR Green SuperMix (Quanta BioSciences). Primer sequences are available upon request from the corresponding author. The mRNA values were normalized to the corresponding GAPDH mRNA.

### Statistics

Data are presented as mean±standard error of the mean (s.e.m.). Differences between experimental groups were tested for statistical significance using unpaired two-tailed Student's *t*-test. *P*-values <0.05 were considered significant.

### Western blot detection

Primary antibodies were diluted (as described in supplementary material Table S1) in blocking buffer (5% w/v milk powder, 25 mM Tris HCl pH 7.4, 135 mM NaCl, 3 mM KCl, 0.02% NP-40). The blots were imaged using an Odyssey® Sa Infrared imaging system (IRDye LiCOR secondary antibody).

### Crude membrane preparation

Tissues were equilibrated in ice-cold homogenization buffer (50 mM NaCl, 0.32 M sucrose, 2 mM EDTA, 20 mM HEPES pH 7.4) containing protease inhibitors and homogenized using a Miccra D-1 homogenizer. The cleared supernatant was centrifuged at ∼100,000 ***g*** to yield cytosol and a membrane pellet.

### Membrane solubilization

Membranes were solubilized at 1 mg/ml of total protein in solubilization buffer (1.5% Triton X-100, 0.75% sodium deoxycholate, 0.1% SDS in 10 mM NaCl, 5 mM EDTA, 2.5 mM EGTA and 50 mM Tris HCl pH 7.35) containing protease inhibitors. The solubilized extracts were centrifuged at 50,000 ***g***, the supernatants were precipitated with trichloroacetic acid and acetone washed, and the resultant pellet was resuspended in 1× SDS sample buffer. Unless stated otherwise, the SDS sample buffer contained 100 mM dithiothreitol.

### Glycosidase treatment

Membranes (∼100 µg of total protein) were solubilized and re-suspended in reaction buffer (G1, G5 or G7 as appropriate and supplied as 10× buffers by New England BioLabs) with 0.25% NP-40 in a final volume of 40 µl. 2.5 µl (125 U) of neuraminidase, 1 µl (500 U) of Endo H and 1 µl (500 U) of PNGaseF were used per reaction (37°C for 1 h).

### Lectin binding assay

The resin (agarose-conjugated *Triticum vulgaris* lectin) was incubated with wash buffer (150 mM NaCl, 2 mM EDTA, 2 mM EGTA, 20 mM HEPES pH 6.8) for 5 min, washed five times in equilibration buffer (150 mM NaCl, 5 mM MnCl_2_, 5 mM MgCl_2_, 5 mM CaCl_2_, 20 mM Tris HCl pH 7.4) and once in solubilization buffer. Membranes that had been prepared from rat tissues were solubilized at 1 mg/ml of total protein in solubilization buffer (10 mM NaCl, 1.5% Triton X-100, 50 mM Tris HCl pH 7.35). 400 µl of solubilized membrane extract (400 µg protein) was incubated with ∼50 µl of gravity-settled resin for 5 h at 4°C. The resin was washed six times in wash buffer (150 mM NaCl, 2.5 mM MnCl_2_, 2.5 mM MgCl_2_, 2.5 mM CaCl_2_, 50 mM Tris HCl pH 7.4). The bound proteins were eluted with 1× SDS sample buffer.

### Mal-PEG cell surface labeling assay

The method established by Shen and colleagues (Shen et al., 2007) was adapted as follows: Transfected HEK293T cells were washed twice with PBS. Cell-surface-exposed cysteines were reduced using 6 mM tris(2-carboxyethyl) phosphine (TCEP) in serum-free Dulbecco's modified Eagle's medium (pH adjusted to 7.0) and incubated at 4°C for 15 min. Cells were washed twice with serum-free DMEM. Maleimide-conjugated polyethylene glycol (Mal-PEG; molecular mass 5000 Da, Iris Biotech GmbH) was purified by gel filtration on a PD-10 column. 500 µl of 5 mM Mal-PEG solution was used per well of a 6-well multiwell cell culture plate. After 30 min at 4°C, two washes in serum-free DMEM and quenching with 5 mM *N*-Ethylmaleimide (NEM), cells were re-suspended in solubilization buffer (500 mM 6-aminohexanoic acid, 1 mM EDTA, 50 mM imidazole HCl pH 7.0) containing 2.5% w/v digitonin and 5 mM NEM. The lysate was supplemented with 5× SDS-PAGE sample buffer (without DTT). For mouse hearts, the perfusion buffer was saturated with 100% oxygen. Following an equilibration period of 2 min at 37°C, cell-surface-exposed cysteines were reduced using 6 mM TCEP in perfusion buffer (pH adjusted to 7.4) for 6 min at 23°C followed by a 2 min wash. The heart was subsequently perfused with 5 mM Mal-PEG in perfusion buffer for 6 min and quenched by NEM (5 mM) for 5 min.

### Recombinant expression of proteins and purification from *E. coli*

The bait proteins used for binding assays were purified as described previously by Yuan and colleagues (Yuan et al., 2003).

### Binding assays

Purified bait proteins were phosphorylated by using 5 ng of recombinant PKA per reaction in reaction buffer (150 mM KOAc, 5 mM Mg(CH_3_COO)_2_, 2% glycerol, 1 mM EDTA, 20 mM HEPES pH 7.4 and protease inhibitor) and incubating with an ATP regeneration system (10 mM phosphocreatine, 0.5 mM ATP, 0.5 mM GTP, 50 µg/ml creatine phosphokinase) for 6 h. Before the addition of PKA, 5 µM of protein kinase A inhibitor [PKI (5–24); Santa Cruz Biotechnology] was used. Cytosol or total membranes containing 65 µg of protein per reaction were added as indicated. Bait proteins (2.5 µg per reaction) were immobilized on IgG Sepharose following phosphorylation and washed five times with reaction buffer. An equimolar concentration of bait to 14-3-3ζ (tagged with maltose binding protein, MBP) or bait to recombinant COPI was added, incubated for 6 h, washed five times with reaction buffer and eluted with the R18 peptide (PHCVPRDLSWLDLEANMCLP, concentration 100 µM) for 14-3-3 binding or with 1× SDS sample buffer (without DTT) for the COPI binding assay. The COPI coat was prepared as described previously (Sahlmüller et al., 2011). Purification of PKA has also been described previously (Mant et al., 2011).

### Phos-tag PAGE

Phos-tag acrylamide (NARD Institute) was used as per the manufacturer's instructions (stock concentration 5 mM).

### *In vitro* phosphorylation of membranes

Crude membranes were washed twice in stripping buffer (500 mM KCl, 5 mM EDTA, 5 mM EGTA, 50 mM Tris pH 7.4 with protease inhibitors), then resuspended in phosphorylation buffer (150 mM NaOAc, 5 mM Mg(CH_3_COO)_2_, 20 mM Tris-OAc, pH 7.4), and phosphorylated using recombinant purified PKA (5–10 ng/reaction) in the presence of an ATP regeneration system or treated with calf intestinal alkaline phosphatase (2 U) in the presence of PKI (5 µM).

### Immobilized metal affinity chromatography

Crude membranes were solubilized at 1 mg/ml for 30 min at 4°C in Complexiolyte buffer 71 (Logopharm) and then centrifuged at 50,000 ***g***. The extracts, containing ∼75 µg of total protein, were diluted 1∶5 in Phos-tag–agarose binding and wash buffer, and incubated with 7 µg of the indicated antibodies (catalog numbers 2729 and 9624, Cell Signaling Technology) for 30 min at 4°C before use with the Phos-tag matrix. Phos-tag–agarose (NARD Institute) was used as per manufacturer's instructions.

### Immunoprecipitation

10 µg of affinity purified rabbit antibodies (catalog number 2729 and 9624, Cell Signaling Technology) were immobilized on Dynabeads (Protein G) according to the manufacturer's instructions (Life Technologies). Crude mouse heart membranes were solubilized in Complexiolyte buffer 71 at 1 mg/ml for 30 min at 4°C and then centrifuged at 50,000 ***g*** for 15 min). The extracts, containing ∼100 µg of total protein per reaction, were diluted 1∶5 in immunoprecipitation binding and wash buffer [150 mM KCl, 5 mM MgCl_2_, 20 mM Tris HCl, pH 7.4, including protease inhibitors (complete EDTA free, Roche) and the PhosSTOP phosphatase inhibitor cocktail (Roche)], and incubated with the affinity matrix for 30 min at 4°C. Following four washes, the bound proteins were eluted with SDS sample buffer (without DTT).

### Electrophysiology

Inside–out excised membrane patches were voltage-clamped at −50 mV (pipette voltage, +50 mV). Bath (intracellular) and pipette (extracellular) solution had the following composition: 140 mM KCl, 10 mM HEPES, 1 mM EGTA, pH 7.3 (*K*_int_ solution). The working concentrations were 100 µM ATP plus 5 mM Mg^2+^; 300 µM diazoxide or pinacidil. Data are presented as stimulated *I*_rel_ (relative current amplitude in diazoxide or pinacidil, normalized to maximum K_ATP_ current in zero ATP). Data were acquired using the pClamp 8.2 software suite (Axon Instruments) and analyzed using ClampFit and Microsoft Excel software. Data from myocytes that had been pre-treated with 10 µM isoproterenol and 10 µM rolipram for 1–2 h (ISO) were recorded in *K*_int_ solution under the same conditions as described above.

### Optical measurements of action potential duration

Isolated heart preparations were performed as described previously (Glukhov et al., 2010). After isolation, cannulation, motion suppression and dye staining, the preparations were equilibrated for an additional 5–10 min before imaging of control measurements during spontaneous rhythm and ventricular pacing. Hearts were paced at the lateral right ventricular epicardium, the pacing current was twice the diastolic pacing threshold. After control measurements, ISO and ROL (at a final concentration of 10 µM each) were introduced to both superfusion and perfusion lines. Sinus-driven and ventricular-paced recordings were obtained at 5 min intervals for 20 min, glibenclamide (10 µM) was then added to the Tyrode's solution containing ISO and ROL. A customized Matlab-based computer program (Laughner et al., 2012) was used to analyze optical signals, which were filtered using a 3×3 pixel spatial filter and a 0–175 Hz finite impulse response filter. Activation times at the maximum first derivative (dV/dt_max_) of optical action potentials were calculated using normalized optical signals. APD was measured as the interval from activation time to 80% repolarization (APD80%) during continuous pacing for each pixel and then averaged throughout the ventricle. Values are expressed as means±s.e.m. unless otherwise stated. Statistical analysis was performed using one-way ANOVA followed by Tukey's post hoc comparison of means. A value of *P*<0.05 was considered statistically significant.

## Supplementary Material

Supplementary Material
