# Supplementary material for: Tuning the electrical properties of the heart by differential trafficking of KATP ion channel complexes
Source: J Cell Sci. 2014 May 1;127(9):2106–19. doi: 10.1242/jcs.141440 (PMC4004980; doi:10.1242/jcs.141440)
Supplement: Supplementary Material [file supp_127_9_2106__index.html]

Tuning the electrical properties of the heart by differential trafficking of KATP ion channel complexes — Supplementary Material 

# Tuning the electrical properties of the heart by differential trafficking of KATP ion channel complexes

## JCS141440 Supplementary Material

**Files in this Data Supplement:**

- **Supplementary Material**
