# Supplementary material for: Tuning the electrical properties of the heart by differential trafficking of KATP ion channel complexes
Source: J Cell Sci. 2014 May 1;127(9):2106–19. doi: 10.1242/jcs.141440 (PMC4004980; doi:10.1242/jcs.141440)
Supplement: Supplementary Material [file supp_127.9.2106_JCS141440.pdf]

Figure-S1

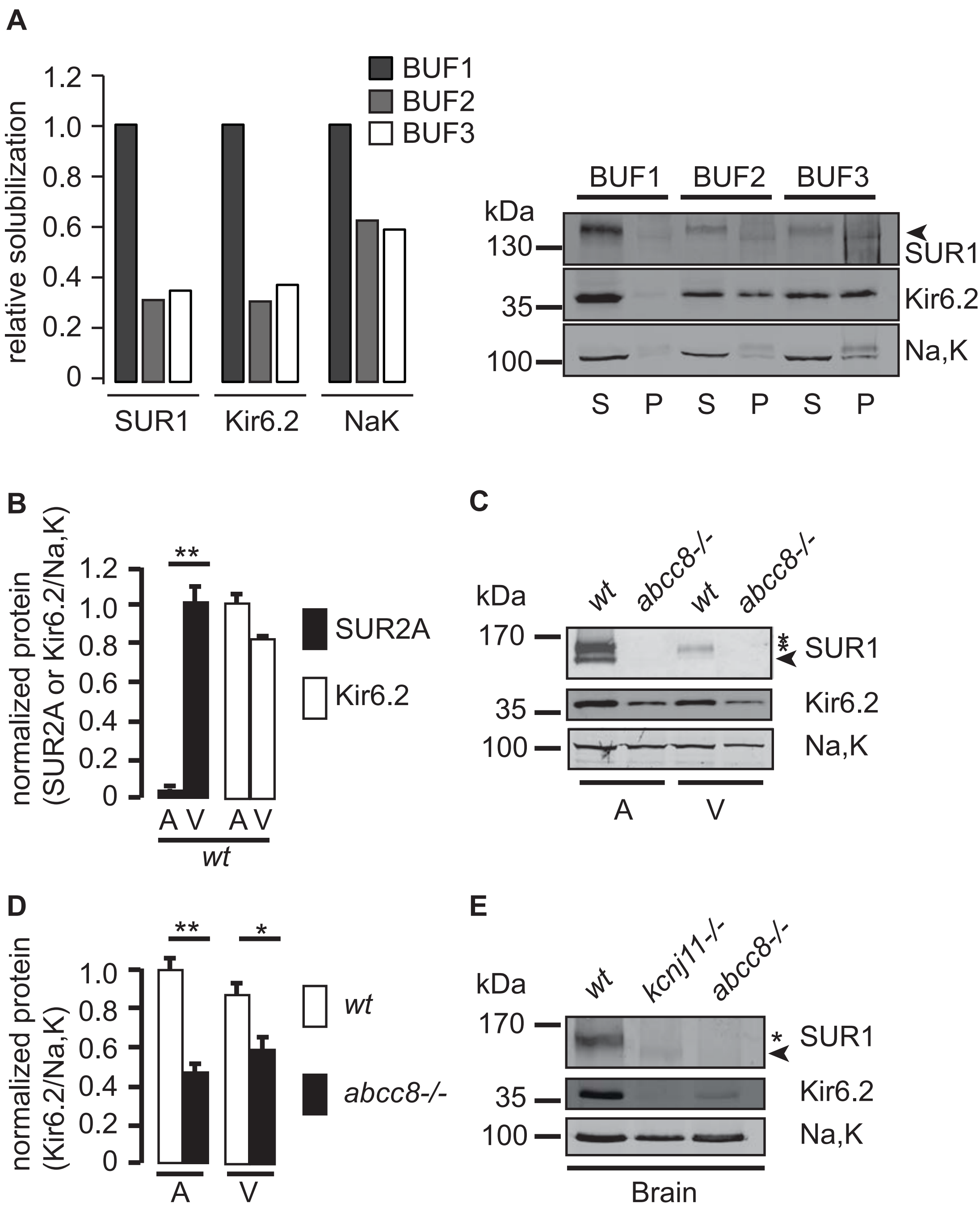
A
BUF1
BUF2
BUF3
relative solubilization
1.2
1.0
0.8
0.6
0.4
0.2
0
SUR1
Kir6.2
NaK
kDa
130
35
100
SUR1
Kir6.2
Na,K
S P S P S P
B
normalized protein (SUR2A or Kir6.2/Na,K)
**
SUR2A
Kir6.2
A V A V
wt
C
wt
abcc8-/-
wt
abcc8-/-
kDa
170
35
100
SUR1
Kir6.2
Na,K
A
V
D
normalized protein (Kir6.2/Na,K)
**
*
wt
abcc8-/-
A
V
E
wt
kcnj11-/-
abcc8-/-
kDa
170
35
100
SUR1
Kir6.2
Na,K
Brain

Figure-S2

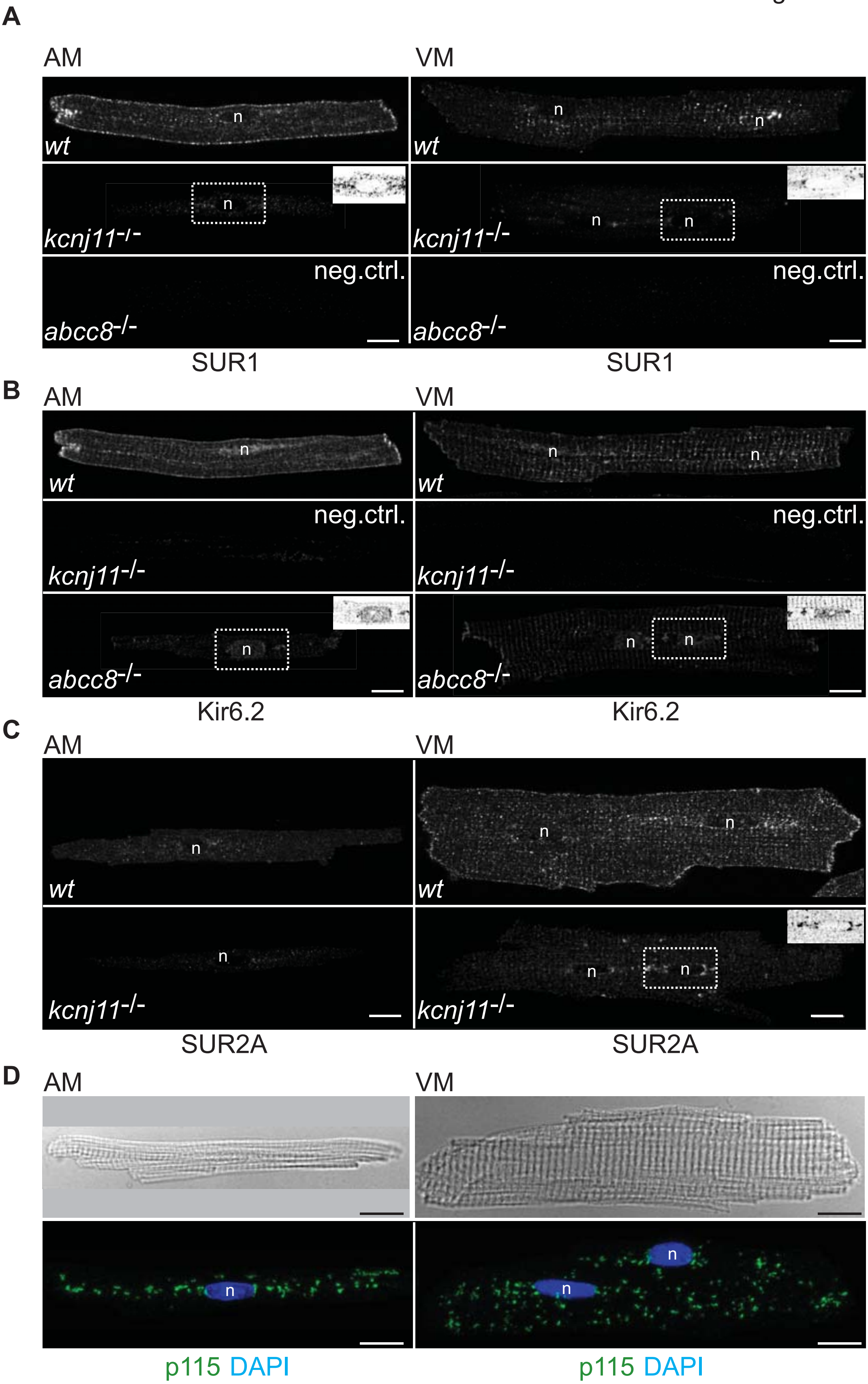

A
AM
VM
n
wt
kcnj11-/-
neg.ctrl.
abcc8-/-
SUR1
SUR1
B
AM
VM
Kir6.2
Kir6.2
C
SUR2A
SUR2A
D
p115 DAPI
p115 DAPI

Figure-S3

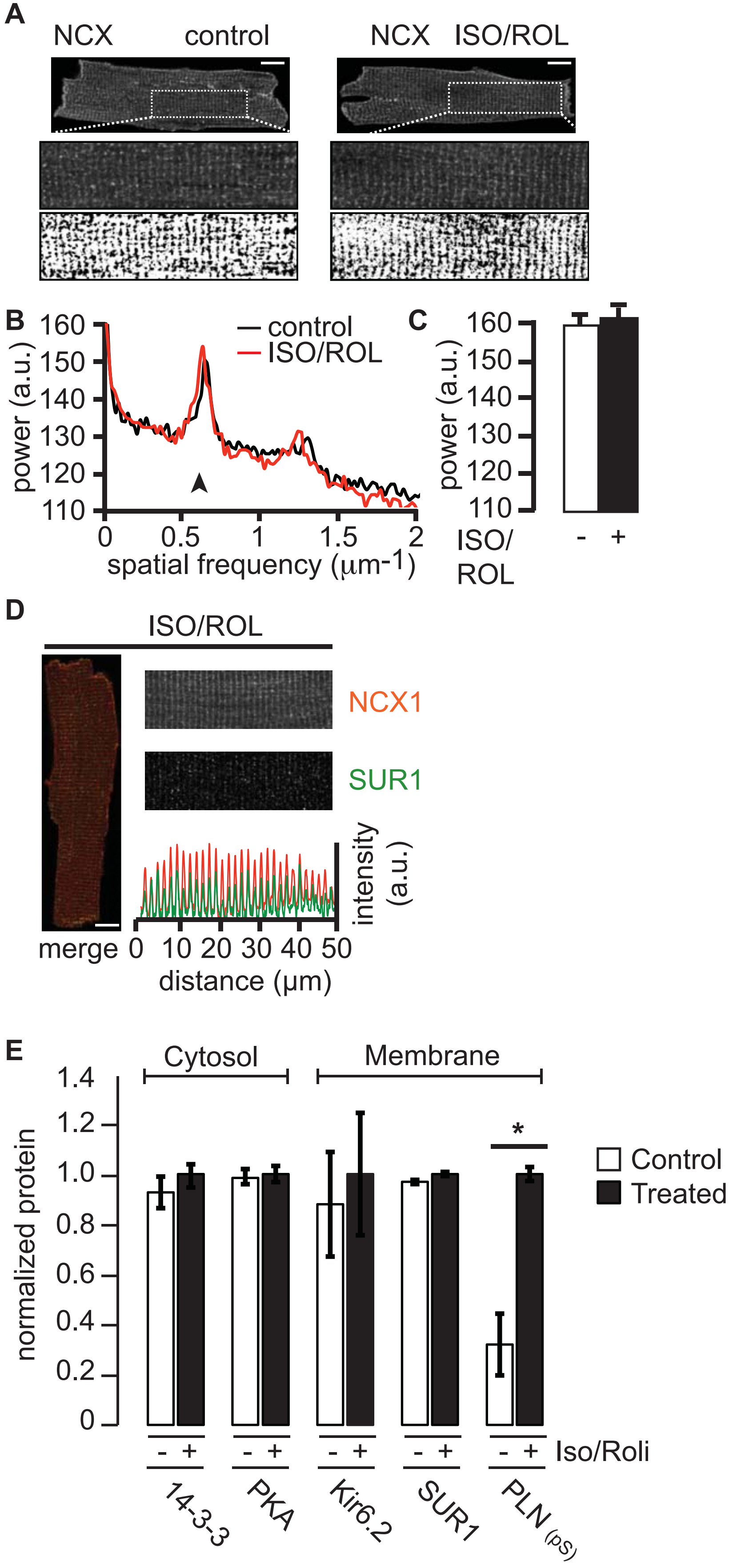

A
NCX control
NCX ISO/ROL
B
control
ISO/ROL
power (a.u.)
spatial frequency (µm-1)
C
power (a.u.)
ISO/ ROL
D
ISO/ROL
NCX1
SUR1
intensity (a.u.)
merge
distance (µm)
E
Cytosol
Membrane
normalized protein
Control
Treated
Iso/Roli
14-3-3
PKA
Kir6.2
SUR1
PLN (pS)

Figure-S4

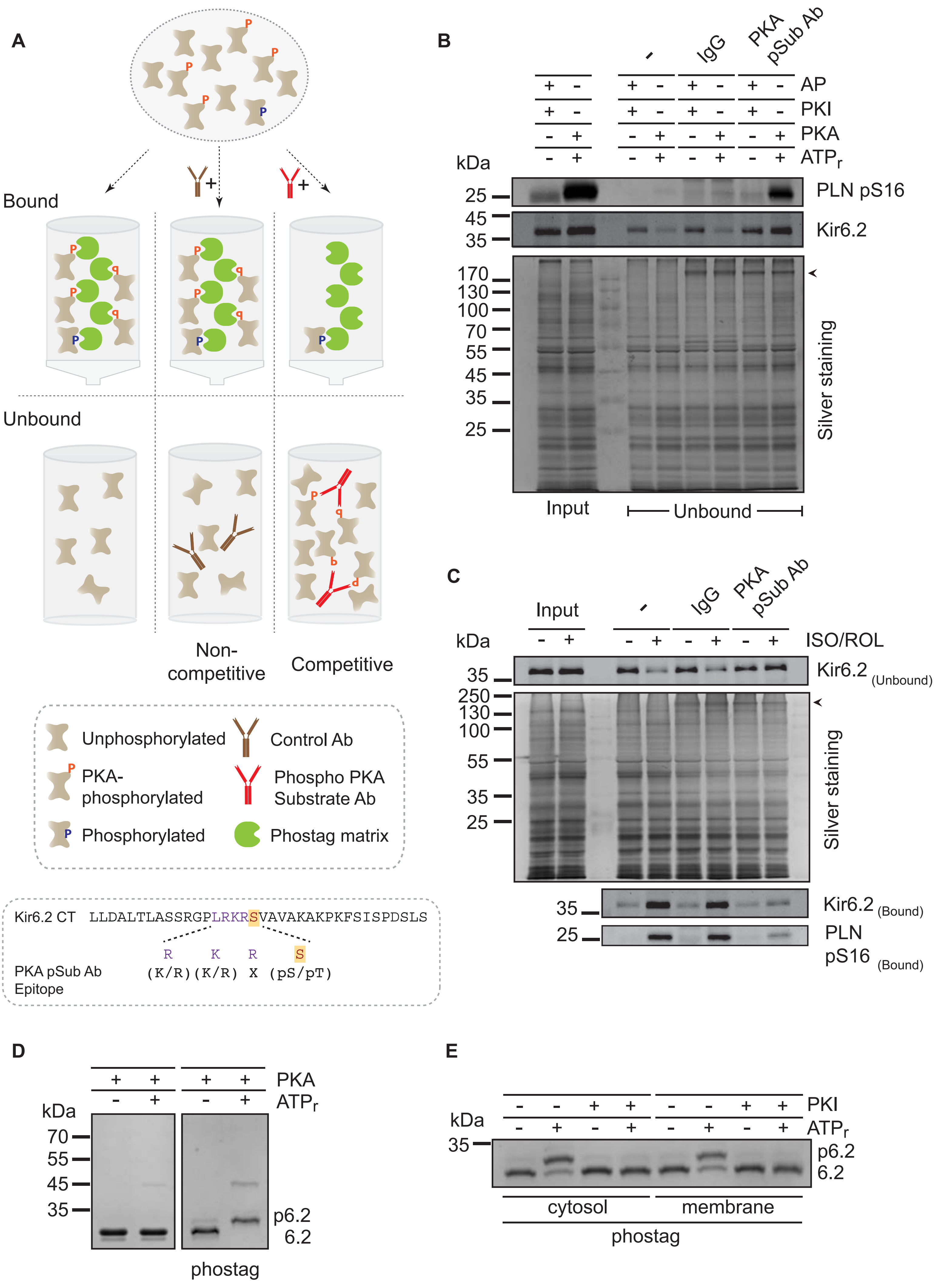
A
Bound
Unbound
Non-competitive
Competitive
Unphosphorylated
Control Ab
PKA-phosphorylated
Phospho PKA Substrate Ab
Phosphorylated
Phostag matrix
Kir6.2 CT LLDALTLASSRGPLRKRSVAVAKAKPKFSISPDSLS
R K R S
PKA pSub Ab Epitope (K/R)(K/R) X (pS/pT)
B
- IgG PKA pSub Ab
AP
PKI
PKA
ATPr
kDa
PLN pS16
Kir6.2
Silver staining
Input
Unbound
C
Input - IgG PKA pSub Ab
ISO/ROL
kDa
Kir6.2 (Unbound)
Silver staining
Kir6.2 (Bound)
PLN pS16 (Bound)
D
PKA
ATPr
kDa
p6.2
6.2
phostag
E
PKI
ATPr
kDa
p6.2
6.2
cytosol
membrane
phostag

Figure-S5

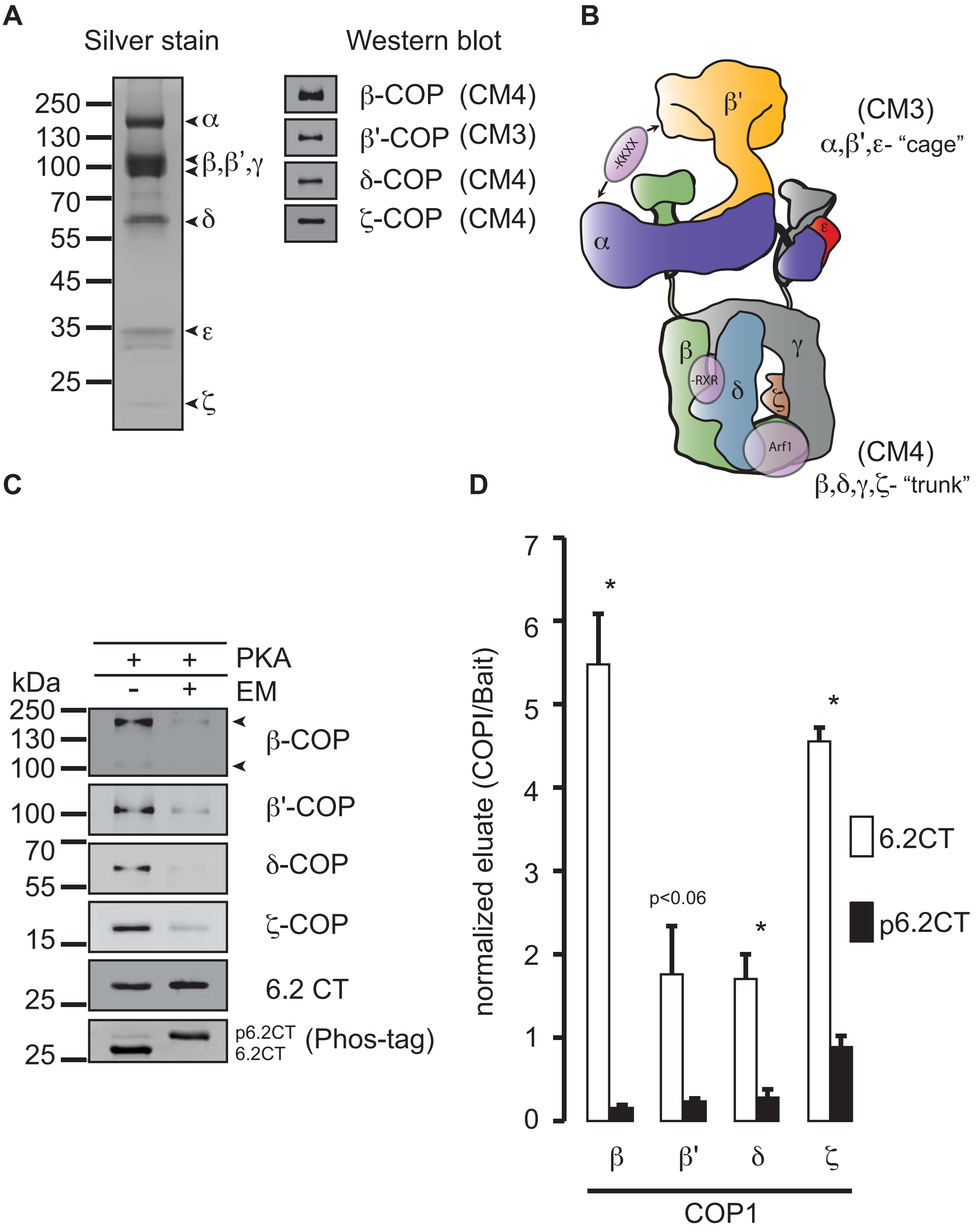
A
Silver stain
Western blot
250
130
100
70
55
45
35
25
α
β,β',γ
δ
ε
ζ
β-COP (CM4)
β'-COP (CM3)
δ-COP (CM4)
ζ-COP (CM4)
B
β'
-KKXX
α
(CM3)
α,β',ε- "cage"
ε
β
γ
-RXR
δ
ζ
Arf1
(CM4)
β,δ,γ,ζ- "trunk"
C
kDa
+ + PKA
- + EM
250
130
100
β-COP
100
β'-COP
70
55
δ-COP
15
ζ-COP
25
6.2 CT
25
p6.2CT
6.2CT
(Phos-tag)
D
normalized eluate (COPI/Bait)
0
1
2
3
4
5
6
7
*
p<0.06
*
*
6.2CT
p6.2CT
β
β'
δ
ζ
COP1

Table-S1 Antibody List											
	Antigen		Clone/ Name	Spec.	Source	Cat. No.	Lot No.	Conc.	Dil. (WB)	Dil. (IF)	Dil. (EM)
1	14-3-3	a)	H-8	Mouse	Santa Cruz Biotechnology, inc.	SC-1657	#E1011	200 μg/ml	1:1000		1:20
		b)	K-19	Rabbit	Santa Cruz Biotechnology, inc.	SC-629	#D1406, #I1411, #B2712	200 μg/ml	1:1000	1:500	
		c)	T-16	Rabbit	Santa Cruz Biotechnology, inc.	SC-1020	#I1008	200 μg/ml	1:1000		
2	Kir6.2		#3	Guinea Pig	Self-made	-	-	Serum	1:1000	1:500	
3	Na+K+ATPase		C464.6	Mouse	Santa Cruz Biotechnology, inc.	SC-21712	#G3004	200 μg/ml	1:1000		
4	SUR1		N289/16	Mouse	Neuromab	75-267	#4492AK44	1.03 mg/ml	1:500		
						73-267	#4375VA10	26.9 μg/ml	1:25	1:10	
5	SUR2A		N319A/ 14	Mouse	Neuromab	75-296	#4493AK38	1.01 mg/ml	1:200		
						73-296	#4376VA13	22 μg/ml	1:15	1:5	
6	NCX1		-	Rabbit	Swant	11-13	#28	*	1:1000	1:500	
7	Nav1.5		-	Rabbit	Alomone Labs	ASC-005	AN2502	0.8 mg/ml	1:1000		
8	β1- Adrenergic receptor		V-19	Rabbit	Santa Cruz Biotechnology, inc.	SC-568	#C0111	200 μg/ml	1:1000		
9	β- Dystroglycan		4F7	Mouse	Santa Cruz Biotechnology, inc.	SC-33702	#J0808	200 μg/ml	1:1000		
10	p115		-	Mouse	BD Transduction Laboratories	611434/ 612260	#73424/ #28136	250 μg/ml		1:500	1:80
11	GAPDH		-	Mouse	Biotrend Chemikalien GmbH	5G4-6C5	#11/06-G4-C5/ #08/07-G4-C5	5.3 mg/ml 12.8 mg/ml	1:40000		
12	Protein- C (Epitope)		HPC4	Mouse	Roche	11814508001	-		1:2500		
13	β-COP (Beta)		B:1.2	Rabbit	Felix Wieland	-	-	Serum	1:2000		
			M3A5	Mouse	Sigma	G-2279	G-2279	Ascite Sup.	1:500		
14	β′-COP (Beta-Prime)		CIPL	Rabbit	Felix Wieland	-	-	Serum	1:3000		

15	δ-COP (Zeta)	#877	Rabbit	Felix Wieland	-	-	Serum	1:1000		
16	ζ1-COP (Delta)	-	Rabbit	Felix Wieland	-	-	Serum	1:3000		
17	ζ2-COP (Delta)	-	Rabbit	Felix Wieland	-	-	Serum	1:2500		
18	pSer16 Phospholamban	-	Rabbit	Badrilla Ltd.	A-010-12	#1010-01	*	1:5000		
19	Phospholamban	2D12	Mouse	Abcam	ab2865			1:2500		
20	Rabbit Normal IgG		Rabbit	Cell Signaling Technology	#2729	6	1 mg/ml			
21	Phospho-PKA substrate	100G7E	Rabbit	Cell Signaling Technology	#9624	16				

* Reconstituted as recommended

Table-S2

Protein	Alignment	Topology	Characterisation	Reference
CD74 (Ii p35)	H.s. MHRRRSRSCREDQ	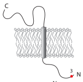	14-3-3 binding site<br>Ser-6 and Ser-8 phosphorylated<br>Cell surface expression upon mutation of Arg-based signal	Kuwana T et al., Proc Natl Acad Sci U S A. 1998 Feb 3;95(3):1056-61<br>Khalil H et al., Int Immunol. 2003 Oct;15(10):1249-63<br>Khalil H et al., J Cell Sci. 2005 Oct 15;118(Pt 20):4679-87
ADAM22	H.s. NGRPRSNS...KRFRPRSNS<br>M.m. NGRPRSNS...KRFRPRSNS<br>B.t. NGRPRSNS...KRFRPRSNS	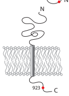	14-3-3 binding site<br>Cell surface expression upon mutation of Arg-based signal	Gödde NJ et al., J Cell Sci., 2006 Aug 15;119 (Pt 16):3296-305.
TMX4	H.s. VEDSLRQRKSQHAD<br>M.m. VEDALRQRKSQNAN<br>B.t. LGDSLRQRRSQHAD	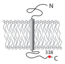	Cell surface expression upon mutation of Arg-based signal	Roth D et al., Biochem J., 2009 Dec 14;425(1):195-205
PLN	H.s. TRSAIRRASTIEM<br>M.m. TRSAIRRASTIEM<br>B.t. TRSAIRRASTIEM	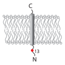	COPI binding site<br>PKA phosphorylation of Ser-16<br>Cell surface expression upon mutation of Arg-based signal	Sharma P et al., PLoS One., 2010 Jul 9;5(7):e11496.
Kir6.1	H.s. HQNSLRKRNSMRRN<br>M.m. HQNSLRKRNSMRRN<br>B.t. HQNSLRKRNSMRRN	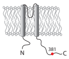	Cell surface expression upon mutation of Arg-based signal	Zerangue N et al., Neuron. 1999 Mar;22(3):537-48.
Kir6.2	H.s. ARGPLRKRSVPMA<br>M.m. SRGPLRKRSVAVA<br>G.g. PRGTIRKRSVKLK	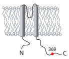	14-3-3 binding site<br>COPI binding site<br>Cell surface expression upon mutation of Arg-based signal	Zerangue N et al., Neuron. 1999 Mar;22(3):537-48<br>Yuan H et al., Curr Biol. 2003 Apr 15;13(8):638-46<br>Heusser K et al., J Cell Sci. 2006 Oct 15;119(Pt 20):4353-63
ENaC (**α**)	H.s. LLRRFRSRYWSP<br>M.m. LLHRFRSRYWSP<br>B.t. LLRRFRSRYWSP	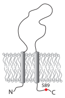	Arg-based signal characterised as an ER-exit signal	Mueller GM et al., J Biol Chem. 2007 Nov 16;282(46):33475-83
KA-2	H.s. RKTSRSRRRRRPGGPS<br>M.m. RKTSRSRRRRRPGGPS<br>B.t. RKTSRSRRRRRPGGPS	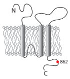	Partial cell surface expression upon mutation of Arg-based signal.<br>Second Arg-based motif in adjacent intracellular loop (580).	Nasu-Nishimura Y et al., J Neurosci. 2006 Jun 28;26(26):7014-21.<br>Ren Z et al., J Neurosci. 2003 Jul 23;23(16):6608-16
NMDA-R1	H.s. LASSFKRRRSSKDT<br>M.m. LASSFKRRRSSKDT<br>G.g. LASSFKRRRSSKDT	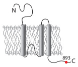	COPI binding site<br>PKC, PKA phosphorylation of Ser-896, Ser-897<br>Cell surface expression upon mutation of Arg-based signal	Scott DB et al., J Neurosci. 2001 May 1;21(9):3063-72<br>Scott DB et al., Neuropharmacology. 2003 Nov;45(6):755-67
TASK1	H.s. GLMKRRSSV<br>M.m. GLMKRRSSV<br>B.t. GLMKRRSSV	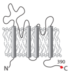	14-3-3 binding site<br>COPI binding site<br>PKA phosphorylation of Ser-392, 393	Rajan S et al., J Physiol. 2002 Nov 15;545(Pt 1):13-26<br>Zuzarte M et al., J Physiol. 2009 Mar 1;587(Pt 5):929-52<br>Mant A et al., J Biol Chem. 2011 Apr 22;286(16):14110-9
TASK3	H.s. RLMKRRKSV<br>M.m. RLHLRRKSI<br>B.t. RLMRRRKSI	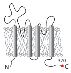	14-3-3 binding site<br>COPI binding site<br>PKA phosphorylation of Ser-373	Rajan S et al., J Physiol. 2002 Nov 15;545(Pt 1):13-26<br>Zuzarte M et al., J Physiol. 2009 Mar 1;587(Pt 5):929-52<br>Mant A et al., J Biol Chem. 2011 Apr 22;286(16):14110-9
SOAT1	H.s. EKMSLRNRLSKSRE<br>M.m. --MSLRNRLSKSGE<br>B.t. EKMSVRKRLSKSSN	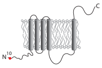	COPI binding site<br>Cell surface expression upon mutation of Arg-based signal	Sharma P et al., PLoS One., 2010 Jul 9;5(7):e11496.
$GABA_BR1$	H.s. SRQQLRSRRHPPT<br>M.m. SRQQIRSRRHPPT<br>B.t. SRQQLRSRRHPPT	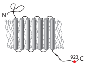	14-3-3 binding site<br>COPI binding site<br>Cell surface expression upon mutation of Arg-based signal	Pagano A et al., J Neurosci. 2001 Feb 15;21(4):1189-202<br>Couve A et al., Mol Cell Neurosci. 2001 Feb;17(2):317-28<br>Brock C et al., Mol Biol Cell. 2005 Dec;16(12):5572-8<br>Margeta-Mitrovic M et al., Neuron. 2000 Jul;27(1):97-106<br>Laffray S et al., EMBO J. 2012 Aug 1;31(15):3239-51
OPRK1	H.s. RMERQSTSRVRNTV<br>M.m. RMERQSTNRVRNTV<br>B.t. RMERQSTSRVRNTV	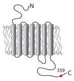	14-3-3 binding site<br>COPI binding site	Li JG et al., J Biol Chem. 2012 Sep 18
GPR15	H.s. EDFARRRKRSVSL<br>M.m. EDFIRRRKRSVSL<br>B.t. EDFARKRKRSVSL	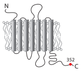	14-3-3 binding site<br>COPI binding site<br>Cell surface expression upon mutation of Arg-based signal	Okamoto Y et al., J Biol Chem. 2011 Mar 4;286(9):7171-81
MDR1	H.s. SRSSLIRKRSTRRS<br>M.m. SGSSLIRRRSTRKS<br>B.t. SRSSLIRRKSTRRS	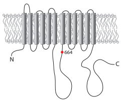	Arg-based signal characterised as an ER-exit signal	Prokopenko O et al., Am J Physiol Cell Physiol. 2009 May;296(5):C1086-97
CFTR1	H.s. PTLQARRRQSVLNL<br>M.m. PTFPGRRRQSVLDL<br>B.t. PTFLGGRRQSVLNL	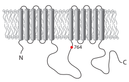	Putative 14-3-3 binding site<br>Putative COPI binding site	Liang X et al., Mol Biol Cell. 2012 Mar;23(6):996-1009
Nav1.8	H.s. GLASGKRRASHGSVF<br>M.m. GLSSGRRRASHSSVF<br>B.t. GLATGRRRASHGSVF	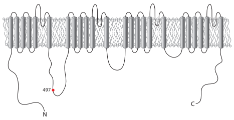	Cell surface expression upon mutation of Arg-based signal	Zhang ZN et al., J Cell Sci. 2008 Oct 1;121(Pt 19):3243-52

SUPPLEMENTARY FIGURE LEGENDS

Fig. S1. Kir6.2 steady-state levels support co-assembly of Kir6.2 and SUR1 in both atria and ventricles. (A) Relative solubilization efficiency of SUR1 and Kir6.2. SDS PAGE analysis of solubilized mouse whole heart membrane fractions ('S' indicates supernatant and 'P' insoluble pellet) using solubilization buffers 'BUF 1-3' (BUF 1: 1.5% Triton-X100, 0.75% Na,deoxycholate, 0.1% SDS in 10 mM NaCl, 5 mM EDTA, 2.5 mM EGTA and 50 mM Tris/HCl pH 7.35; BUF 2: 1.5% Triton-X100 in 10 mM NaCl, 50 mM Tris/HCl pH 7.35; BUF 3: 2.5% w/v Digitonin, 500 mM 6-aminohexanoic acid, 1 mM EDTA, 50 mM imidazole/HCl pH 7.0). Bar graph depicts relative solubilization of SUR1 and Kir6.2 in comparison to the α1 subunit of the Na,K-ATPase 'Na,K' based on densitometric analysis of the blot shown to the right. Signals were normalized to the signal observed in the supernatant after solubilization using BUF1. (B) Quantification of three experiments as shown in Figure 1B illustrating averaged levels of SUR2A (filled bars) and Kir6.2 (open bars) in atria 'A' and ventricles 'V'. Error bars indicate S.E.M. and asterisks significantly lower levels of SUR2A in atria than in ventricles ($p<0.005$). (C) Western blot for the K_{ATP} channel subunits SUR1 and Kir6.2 and α1 subunit of the Na,K-ATPase using mouse cardiac membranes of the indicated genotypes. Filled arrowhead marks the core- and asterisks the complex-glycosylated forms of SUR1. Membranes from mouse atrial 'A' and ventricular tissue 'V' were prepared separately. Blot represents one of three individual experiments. (D) Relative levels of total Kir6.2 protein in atrial and ventricular tissue. Bar graphs summarize three independent experiments performed on membranes pooled from three animals and error bars indicate S.E.M. (E) Western blot for the K_{ATP} channel subunits SUR1 and Kir6.2 and α1 subunit of the Na,K-ATPase using mouse brain membranes of the indicated genotypes. Filled arrowhead marks the core- and asterisk the complex-glycosylated forms of SUR1. The blot is representative of three independent experiments. Membranes from total brain were analyzed because the notion that Kir6.2 and SUR1 co-assemble in neuronal K_{ATP} channels is well established. Hence the analysis confirms reduction of Kir6.2 steady-state levels in the absence of its partner subunit suggesting that Kir6.2 is indeed the partner of SUR1 in cardiac tissue.

Fig. S2. Subcellular localization of SUR1 and Kir6.2 in the absence of the respective partner subunit and antibody controls. Confocal analysis of isolated, immunostained mouse atrial 'AM' or ventricular 'VM' myocytes of the indicated genotype. Anti-SUR1 (A), anti-Kir6.2 (B) and anti-SUR2A (C) immunofluorescence signals are shown. Images obtained from cardiomyocytes not expressing the target protein (*abcc8-/-* in (A) and *kcnj11-/-* in (B); labeled 'neg. ctrl.') demonstrate the specificity of the antibodies used in the relevant cell type by direct comparison using the same immuno-staining conditions. Weak juxtanuclear signals of SUR1/2A or Kir6.2 in cardiac myocytes genotypes lacking the respective partner subunit (knock-out genotypes as indicated) are documented by insets, which show the nucleus ('n' in the large image) and surrounding area as contrast-enhanced, inverted images for the boxed regions of interest. Apart from the juxtanuclear staining, the labeling of the nucleus for Kir6.2 in (B) is considered as unspecific, since it can also be found in the *kcnj11-/-* cells. (D) Projection image of isolated, immunostained wt mouse atrial 'AM' or ventricular 'VM' myocytes stained with an anti-p115 antibody, which labels the cis-Golgi. Nuclei were labeled using DAPI. The corresponding brightfield image of the myocyte is shown above. Projections consist of 16 slices for 'AM' and 15 slices for 'VM' at a slice interval of 0.69 μm. Scale bars 10 μm.

Fig. S3. β-adrenergic stimulation does not affect localization of sodium-calcium exchanger NCX1 to T-tubule membrane invaginations at striations of ventricular myocytes. (A) Confocal analysis of mouse ventricular myocytes immunostained for NCX1 in the absence or presence of stimulation (10 μM ISO and 10 μM ROL). Dashed boxes indicate the magnified (2x) intracellular region of interest showing the direct (middle) and binary inverse contrasted signal (bottom). Scale bar 10 μm. (B) Power spectrum (Fourier analysis) of 17 untreated and 17 treated myocytes; the 1st peak indicates the degree of periodicity of the striated signal (arrowhead). (C) Bar graph summarizing the average change in power at the 1st peak marked in (B); error bars show standard error of the mean (S.E.M). There is no significant difference between treated and untreated cells. (D) Ventricular myocyte co-stained for NCX1 (red) and SUR1 (green). Intensity profiles of depicted sections demonstrate colocalization of SUR1 and NCX1 at T-tubular striations. Scale bar is 10 μm. (E) Quantification of three experiments as shown in Figure 4F illustrating averaged levels

of 14-3-3 proteins (pan-reactive antibody), protein kinase A ‘PKA’, Kir6.2, SUR1 and the serine 16-phosphoform of phospholamban ‘$PLN_{(pS)}$’ in control (open bars) and ISO/ROL-treated (filled bars) ventricular myocytes. Cytosolic proteins were normalized to GAPDH, membrane proteins to the α1 subunit of the Na,K-ATPase. Error bars indicate S.E.M. and asterisk significantly increased levels of phosphorylated phospholamban ($p<0.05$).

Fig. S4. Kir6.2 is phosphorylated upon sustained beta-adrenergic stimulation in vivo. (A) A schematic representation of the experimental setup used to probe the phosphorylation status of a given protein of interest using an Immobilised Metal Affinity Chromatography (IMAC) matrix (Phostag™ agarose). Competitive binding assays that confirm the specificity of the affinity of PKA phosphorylated substrates to the Phos-tag agarose matrix were performed by preincubating the solubilized extracts with either a control antibody (Rabbit IgG) that detects no known antigen or an antibody (PKA pSub Ab) that detects PKA-phosphorylated substrates. Both the bound (eluate) and unbound (flow-through) fractions were analyzed by Western blot detection. The site of PKA phosphorylation (S372) in Kir6.2, most likely recognized by the PKA pSub antibody, has been highlighted. (B) Membranes prepared from wildtype mouse hearts were in vitro phosphorylated using recombinantly purified PKA in the presence of an ATP regeneration system (ATPr) or treated with calf intestinal alkaline phosphatase (AP) in the presence of a PKA inhibitory peptide (PKI). Phosphorylated proteins were enriched using Phos-tag agarose. Antibody competition assays were performed as depicted in (A). The unbound fraction, depleted of phosphorylated proteins, was analyzed by Western blot detection for Kir6.2 and a phosphorylated form of phospholamban (phosphoserine 16). (C) Membranes prepared from wildtype mouse hearts perfused in the presence (+) or absence (-) of 10 μM isoproterenol and 10 μM rolipram were analyzed as depicted in (A). The unbound fraction (depleted of phosphorylated proteins) and the bound fraction (enriched in phosphorylated proteins) were analyzed by Western blot detection as indicated. Filled arrowheads indicate either the IgG or PKA pSub antibody supplementing the solubilized extracts for competitive binding assays. (D) The recombinant catalytic subunit of PKA phosphorylates the C-terminus of Kir6.2 as indicated by altered migration in Phos-tag gel electrophoresis after Coomassie

staining. ‘ATPr’ indicates the use of an ATP regeneration system. (E) Cardiac cytosol and total membranes contain PKA that phosphorylates the C-terminus of Kir6.2. Analysis as in (B), ‘PKI’ indicates protein kinase A inhibitor peptide. (D)

Fig. S5. Quantification of COPI binding to the Kir6.2 C-terminal peptide before and after phosphorylation by PKA. (A) Silver stain of purified recombinant COPI coat and Western blot detection of individual subunits by the indicated antibodies. (B) Schematic depiction of the COPI heptamer with trunk (CM4) and cage (CM3) subcomplexes indicated. Mapped binding sites for C-terminal di-lysine (-KKXX), Arg-based (-RXR) and Arf1 are labeled. (C) Representative blots demonstrating efficient phosphorylation of the Kir6.2 C-terminal peptide and levels of individual COPI subunits in the eluates. (D) Quantification of the fluorescence intensity obtained from Western blot signals reflecting binding of individual COPI subunits to the indicated peptides in three independent experiments. Error bars indicate S.E.M. Asterisk denotes $p<0.05$, non-significant value of $p<0.06$ is indicated for β’-COP.

Table S1. Antibodies used in this study.

Table S2. Synopsis of the characterization of Arg-based signals in membrane proteins. Effects of mutating the Arg-based signal, COPI and 14-3-3 binding or phosphorylation on the indicated cargo protein are summarized. Red line indicates a confirmed COPI binding site and green line a confirmed 14-3-3 binding site.
